# *Prevotella copri* transplantation promotes neurorehabilitation in a mouse model of traumatic brain injury

**DOI:** 10.1186/s12974-024-03116-5

**Published:** 2024-06-04

**Authors:** Nina Gu, Jin Yan, Wei Tang, Zhaosi Zhang, Lin Wang, Zhao Li, Yingwen Wang, Yajun Zhu, Shuang Tang, Jianjun Zhong, Chongjie Cheng, Xiaochuan Sun, Zhijian Huang

**Affiliations:** 1 Department of Neurosurgery, The First Affiliated Hospital of Chongqing Medical University, Chongqing, 400016 China; 2 Department of Neurosurgery, The Second Clinical Medical College of North Sichuan Medical College, Nanchong Central Hospital, Nanchong, China; 3 Emergency Department, Chengdu First People’s Hospital, Chengdu, China; 4 Department of Neurosurgery, Suining Central Hospital, Suining, China

**Keywords:** Traumatic brain injury, *Prevotella copri*, Neurorehabilitation, Gut microbiota, Guanosine, GUO-PI3K/Akt pathway

## Abstract

**Background:**

The gut microbiota plays a critical role in regulating brain function through the microbiome-gut-brain axis (MGBA). Dysbiosis of the gut microbiota is associated with neurological impairment in Traumatic brain injury (TBI) patients. Our previous study found that TBI results in a decrease in the abundance of *Prevotella copri* (*P. copri*). *P. copri* has been shown to have antioxidant effects in various diseases. Meanwhile, guanosine (GUO) is a metabolite of intestinal microbiota that can alleviate oxidative stress after TBI by activating the PI3K/Akt pathway. In this study, we investigated the effect of *P. copri* transplantation on TBI and its relationship with GUO-PI3K/Akt pathway.

**Methods:**

In this study, a controlled cortical impact (CCI) model was used to induce TBI in adult male C57BL/6J mice. Subsequently, *P. copri* was transplanted by intragastric gavage for 7 consecutive days. To investigate the effect of the GUO-PI3K/Akt pathway in *P. copri* transplantation therapy, guanosine (GUO) was administered 2 h after TBI for 7 consecutive days, and PI3K inhibitor (LY294002) was administered 30 min before TBI. Various techniques were used to assess the effects of these interventions, including quantitative PCR, neurological behavior tests, metabolite analysis, ELISA, Western blot analysis, immunofluorescence, Evans blue assays, transmission electron microscopy, FITC-dextran permeability assay, gastrointestinal transit assessment, and 16 S rDNA sequencing.

**Results:**

*P. copri* abundance was significantly reduced after TBI. *P. copri* transplantation alleviated motor and cognitive deficits tested by the NSS, Morris’s water maze and open field test. *P. copri* transplantation attenuated oxidative stress and blood-brain barrier damage and reduced neuronal apoptosis after TBI. In addition, *P. copri* transplantation resulted in the reshaping of the intestinal flora, improved gastrointestinal motility and intestinal permeability. Metabolomics and ELISA analysis revealed a significant increase in GUO levels in feces, serum and injured brain after *P. copri* transplantation. Furthermore, the expression of p-PI3K and p-Akt was found to be increased after *P. copri* transplantation and GUO treatment. Notably, PI3K inhibitor LY294002 treatment attenuated the observed improvements.

**Conclusions:**

We demonstrate for the first time that *P. copri* transplantation can improve GI functions and alter gut microbiota dysbiosis after TBI. Additionally, *P. copri* transplantation can ameliorate neurological deficits, possibly via the GUO-PI3K/Akt signaling pathway after TBI.

**Supplementary Information:**

The online version contains supplementary material available at 10.1186/s12974-024-03116-5.

## Background

Traumatic brain injury (TBI) represents an enormous challenge to public health. More than 50 million people worldwide suffer TBI each year, and it is estimated that approximately half of the world’s population will experience one or more TBI during their lifetime [1]. It is well established that secondary injury is the major cause of TBI-associated brain damage. In the past few decades, much progress has been made in studying the pathological mechanisms of TBI and in developing therapeutic strategies [2, 3]. Targeting the unfavorable lesion microenvironment such as free radicals and neuroinflammation after TBI holds great promise for restoring neural function [4]. Excessive reactive oxidative stress (ROS) production is associated with neuroinflammation and chronic neurodegeneration following TBI [5]. Inhibition of oxidative stress after TBI markedly reduces pro-inflammatory activation of macrophages/microglia with a concomitant increase in anti-inflammatory responses [6].Therefore, anti-oxidative stress treatment helps alleviate neuroinflammation after TBI. Although anti-oxidative stress target and anti-neuroinflammation target are fairly distinct, they are interwoven, interactive and interdependent.

At present, many drugs showed good therapeutic effects in experimental animals, but most of their clinical trials failed, it still remains a challenging task to develop an effective strategy for the treatment of TBI [7]. On the other hand, it has been found that some central nervous system (CNS) diseases, including acute ischemic stroke [8, 9], Alzheimer’s disease [10] and Parkinson’s disease [11], can be treated by regulating the gut microbiome. Likewise, it has been found that TBI can cause changes in the intestinal microbiota, and the intestinal flora changes significantly in number and categories, mainly involving the *pseudorod Bacteria*, *Purpuromonas*, *Firmicutes*, *Proteobacteria* among others [12, 13]. In our previous research, we found that fecal microbiota transplantation can restore gut microbiota dysbiosis and alleviate neurological deficits after TBI [14]. Randall et al. found that the change in *Prevotella* was the most significant microbiota after TBI, and the abundance of *P. copri* in *Prevotella* was significantly reduced by 4.5-fold [15]. However, in-depth studies on how to affect the disease course after TBI by regulating a specific strain of gut microbiota are still lacking. *Prevotella* are Gram-negative anaerobes that have previously been identified as potential key factors in shaping gut function and host health [16]. *Prevotella* can ferment dietary fiber to produce the short chain fatty acid (SCFA) acetate. They can also metabolize dietary fiber and fat to produce succinate [17, 18]. *P. copri* is an abundant member of the human gastrointestinal microbiota. The effect of *P. copri* on human health is currently inconclusive. Its relative abundance may play a positive or negative role in disease [19]. Whether *P. copri* transplantation can have a protective effect on TBI’s neurological function is still in need of further confirmation.

Gut bacteria regulate the central nervous systems through dynamic bidirectional communication along the ‘gut–brain axis’ in a microbial metabolites-dependent manner [20]. Guanosine (GUO) is an intermediate product of nucleotide metabolism, which is produced by the decomposition of GMP by the 5 ‘-nucleotidase (5’ -NT) of intestinal bacteria, and is absorbed into the blood by intestinal epithelial cells. Then GUO is transported through the Nucleotide transporter on the surface of cerebral microvascular endothelial cells and enters the brain tissue rapidly through the blood brain barrier (BBB) [21, 22]. Guanosine plays an important neuroprotective role in CNS neural injury. Guanosine is neuroprotective against focal cerebral ischemia by inhibiting microglia/macrophage activation and mediating anti-inflammatory response that ameliorates neural injury [23]. Guanosine inhibits LPS-induced pro-inflammatory response and oxidative stress in hippocampal astrocytes [24]. Previous studies have confirmed that GUO can alleviate oxidative stress after TBI by activating PI3K/Akt pathway in astrocytes and hippocampus cultured in vitro [25]. GUO also reduces mitochondrial oxidative stress damage caused by inhibition of mitochondrial complex by activating PI3K/Akt pathway in neuroblastocytes [26].

In this study, we hypothesized that *P. copri* transplantation could relieve the neurological deficits in a TBI mouse model in a GUO-dependent manner. GUO might be upregulated by *P. copri* treatment and thereafter activating the antioxidant PI3K/Akt pathway.

## Materials and methods

### Animals

Adult C57BL/6J mice (18–22 g, 8 weeks old) were purchased from the Laboratory Animal Center of Chongqing Medical University (Chongqing, China). The groups were housed separately. Mice were kept under standard conditions (temperature, 22 ± 2 °C; humidity, 55 ± 10%) with a 12:12 light/dark cycle. Food and water were available ad libitum. All procedures were performed in a specific pathogen-free (SPF) environment, and all tools and materials were sterilized with 75% alcohol. The type of bedding was poplar wood shavings (Chongqing Tengxin Biotechnology Co., LTD), the food was standard chow(Beijing Huafukang Biotechnology Co., LTD), the water was autoclaved purified water.All procedures on animals were approved by the Institutional Animal Care and Use Committee of Chongqing Medical University(approval No. 2,019,335) and carried out in accordance with ARRIVE guidelines [27].

### CCI model

A standard protocol and a controlled cortical impact (CCI) device (TBI-0310, Precision Systems and Instrumentation, Fair fax, VA, USA) were utilized to induce brain injuries as described previously [28]. Mice were anesthetized with isoflurane (3% induction, 2% maintenance) when they underwent any surgeries. Briefly, a circular craniotomy was performed in the right sensorimotor cortex and above the hippocampus (center of the impact: A/P, − 2.00 mm; M/L, 2.50 mm from bregma). Following craniotomy, a CCI model was established with a TBI-0310 TBI model system, and the impact parameters were set as follows: 5 m/s velocity, 100 ms dwelling time, 2 mm depth and 3 mm diameter impactor. A pneumatic impactor was used to provide the power for impact (Jun-Air Model 3–4). Sham control group mice underwent only craniotomy without CCI. Following the injury, the hole was sealed with bone wax, and the skin incision was sutured. Finally, the mice were kept on an electric blanket (69,001, RWD Life Science, China) to maintain their body temperature at 37.5 ± 0.5 °C until completely awakening (about 3–4 h).

### Pharmacological intervention

In accordance with a previous study, antibiotics intervention was used to remove intestinal flora and minimize the difference in intestinal flora among the experimental groups of mice before the CCI operation or *P. copri treatment* [29]. Before the CCI operation, we administered antibiotics to adult mice (6 weeks of age) in all groups through drinking water containing 0.2 g/L ampicillin (Macklin, China), neomycin (Macklin, China), metronidazole (Macklin, China) and 0.1 g/L vancomycin (Macklin, China) daily for 2 weeks. At 2 h after TBI, the TBI + Vehicle group received 0.1 ml of 10% glycerol in PBS solution, while the TBI + *P. copri* group received a dose of 0.1 ml containing 5 × 10^6^ CFU/mL *P. copri* in 10% glycerol in PBS via intragastric gavage [30]. Then, the same perfusion method was performed for 7 consecutive days (every morning at 9–10 a.m.). As previously described, GUO (Macklin, China) solution dissolved in 0.9% saline was maintained at 37 °C before injections to prevent any drug precipitation [31]. GUO (7.5 mg/kg) was intraperitoneally administered (1 mL/kg) to mice at every day for 7 days (every morning at 9–10 a.m.) and the first administration was performed 2 h after TBI. LY294002 (Macklin, China) solution dissolved in 5% dimethyl sulfoxide and 25 mg/kg LY294002 were administered via intracerebroventricular injection 30 min before TBI [32].

### Experimental protocols

**Fig. 1:**
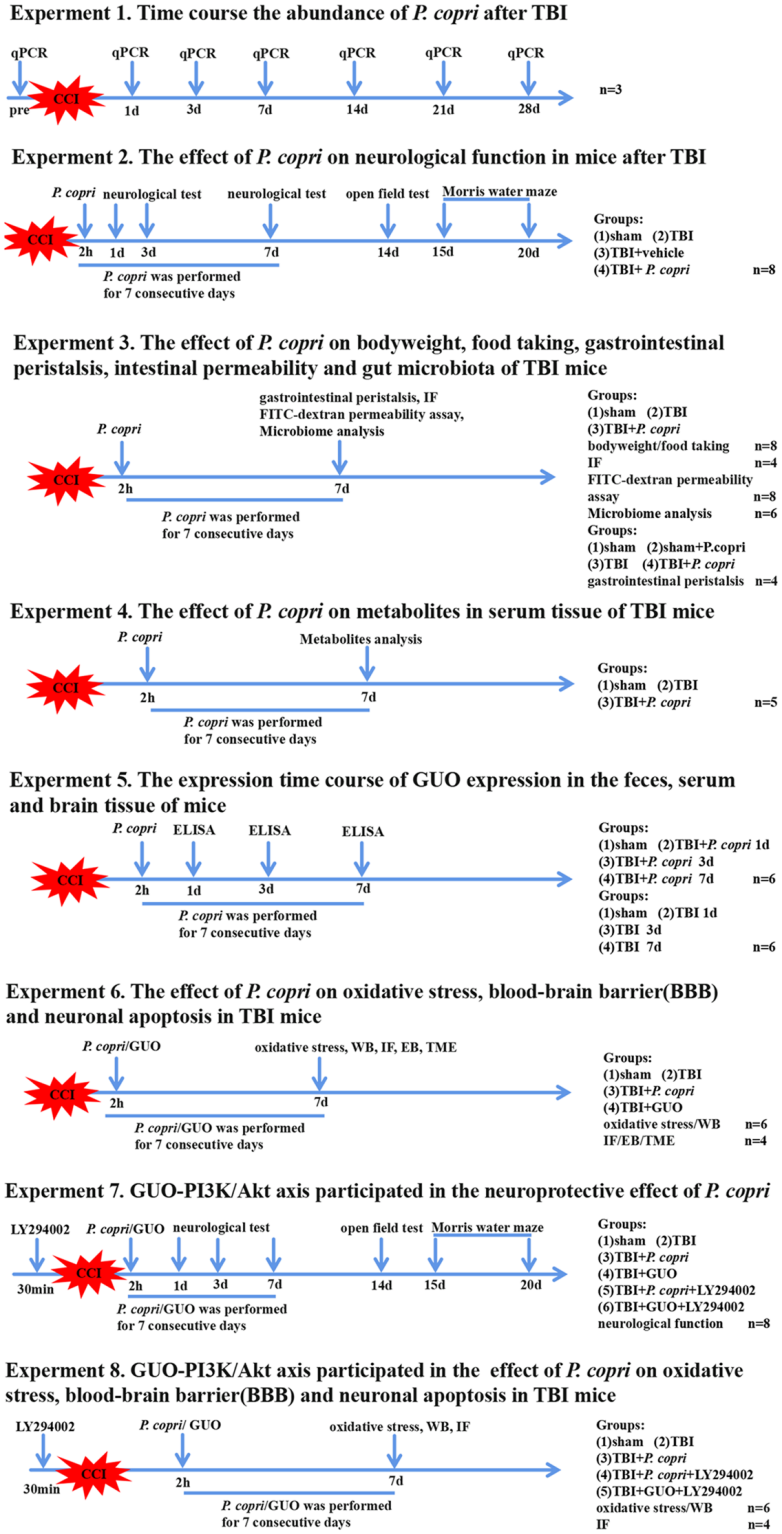
Schematic diagram of the different experimental protocols and setups used in this study. *P. copri*, *Prevotella copri*; CCI, controlled cortical impact; TBI, traumatic brain injury; WB, western blot; IF, immunofluorescence; GUO, guanosine; BBB, blood-brain barrier; TME, transmission electron microscopy

All experimental protocols and setups are shown in Fig. 1.

### Experiment 1

To determine the time course of *P. copri* abundance in the feces of mice after TBI. Feces sample from a group of three mice feces were collected at different time periods: before TBI, 1 d, 3 d, 7 d, 14 d, 21 d, and 28 d after TBI. A quantitative real-time PCR (QPCR) assay was performed to evaluate the changes in *P. copri* abundance.

### Experiment 2

To evaluate the effects of *P. copri* on neurological function in mice after TBI, 32 mice were randomly divided into four groups: sham group, TBI group, TBI + Vehicle group, TBI + *P. copri* group (*n* = 8 per group). All mice were subjected to Neurological Severity Scores (NSS) and wire grip tests to evaluate short-term neurological function. Subsequently, all mice were subjected to the Morris water maze (MWM) and the open field test (OFT) to evaluate learning and memory function. The OFT and MWM were performed up to 2 weeks after TBI.

### Experiment 3

To evaluate the effects of *P. copri* transplantation on body weight, food intake, intestinal permeability and microbiome analysis in TBI, mice were randomly divided into three groups: sham group, TBI group, TBI + *P. copri* group (*n* = 18 per group). We randomly recorded body weight and food consumption of 8 mice in each group. Four mice were subjected to immunofluorescence staining, 8 mice were used for FITC-dextran permeability assay, and 6 mice were used for microbiome analysis. To evaluate the effects of *P. copri* transplantation on gastrointestinal motility, mice were randomly divided into four groups: sham group, sham + *P. copri* group, TBI group, TBI + *P. copri* group (*n* = 4 per group), and 4 mice in each group were used for gastrointestinal transit assessment.

### Experiment 4

To estimate the effects of *P. copri* on serum metabolites in TBI mice, 15 mice were randomly divided into three groups: the sham group, the TBI group, and the TBI + *P. copri* group (*n* = 5 per group). After 7 days of *P. copri* treatment, serum from each mouse was collected for metabolite analysis.

### Experiment 5

To evaluate the time course of GUO presence in the feces, serum and brain tissue of mice at the injury site after *P. copri* treatment, 48 mice were used in this experiment: [1] 24 mice were continuously treated with *P. copri* for 7 days after TBI and randomly divided into four groups: sham, TBI + *P. copri* 1d, TBI + *P. copri* 3d, and TBI + *P. copri* 7d (*n* = 6 per group); [2] as a control, 24 mice were not transplanted with *P. copri* after TBI and were randomly divided into four groups: sham, TBI 1d, TBI 3d, and TBI 7d (*n* = 6 per group). ELISA was performed to evaluate the changes in GUO presence.

### Experiment 6

To investigate the effect of *P. copri* transplantation on oxidative stress, blood-brain barrier (BBB) permeability and neuronal apoptosis in TBI mice, mice were randomly divided into four groups: sham group, TBI group, TBI + *P. copri* group, and TBI + GUO group. For oxidative stress study, every 6 mice in each group were assigned to Catalase (CAT) measurements, Superoxide dismutase (SOD) measurements, Reactive oxygen species (ROS) measurements, Oxidized glutathione (GSSG) and Reduced glutathione (GSH)/GSSG ratio measurements. For BBB permeability study, every 6 mice were assigned to Western blot analysis; 4 mice were assigned to immunofluorescence staining of Occludin; 6 mice were assigned to Evans blue (EB) assay, 4 mice were assigned to EB fluorescence observation; and 4 mice were assigned to transmission electron microscopy examination. For neuronal apoptosis study, 4 mice were assigned to immunofluorescence staining.

### Experiment 7

To determine whether the GUO-PI3K/Akt pathway plays a role in the neuroprotective effects of *P. copri* transplantation, mice were randomly divided into six groups: sham group, TBI group, TBI + *P. copri* group, TBI + GUO group, TBI + *P. copri* + LY294002 group, and TBI + GUO + LY294002 group. Western blot analysis was performed to detect key proteins (p-PI3K, p-Akt) in the GUO-PI3K/Akt pathway using 6 mice per group. For behavioral tests, 8 mice were assigned to each group as carried out in Experiment 2.

### Experiment 8

To determine whether the GUO-PI3K/Akt pathway is involved in the effects of *P. copri* on oxidative stress, blood-brain barrier (BBB), and neuronal apoptosis in TBI mice, mice were randomly divided into five groups: sham group, TBI group, TBI + *P. copri* group, TBI + *P. copri* + LY294002 group and TBI + GUO + LY294002 group. For oxidative stress study, every 6 mice in each group were assigned to SOD measurements and ROS measurement. For BBB permeability study, every 6 mice were assigned to Western blot analysis of ZO-1 and Occludin; 4 mice were assigned to immunofluorescence staining of Occludin. For neuronal apoptosis study, 6 mice were assigned to Western blot analysis of Bcl-2 and Bax.

### Real-time quantitative PCR (qPCR)

Total DNA from mouse feces was extracted using TRIzol (Invitrogen, Carlsbad, CA, USA) based on the manufacturer’s protocol. Confirmation of colonization was achieved with pcopri2F_pcopri2R (F: ACCACTTGGGGATAACCTTG, R: TACATGCAAAAAGCCTCACGAGGC). Detailed procedures are provided in Supplemental File.

### Neurobehavioral assessment

The investigators were blinded to the experimental groups for all tests. Tests were conducted in a quiet room at 22 ± 2 °C and 55 ± 10% relative humidity.

### NSS scores and wire grip test

NSS analyses and wire grip tests were performed in accordance with previous research [33]. The Neurological Severity Scores (NSS) measured general behavior, alertness, balance and motor ability using ten different tasks. Two researchers who were blinded to each group used NSS to assess the neurologic deficits on 1 day pre-injury and on days 1, 3 and 7 after TBI. One point was obtained for each failed task. Zero points represented the minimum deficit, and ten points represented the maximum deficit. Table 1 in supplemental file shows details of the NSS score.

**Table 1:** Neurological severity score (NSS) for head-injured mice

Task	NSS
Presence of mono- or hemiparesis	1
Inability to walk on a 3-cm-wide beam 1	1
Inability to walk on a 2-cm-wide beam	1
Inability to walk on a 1-cm-wide beam	1
Inability to balance on a 1-cm-wide beam	1
Inability to balance on a round stick (0.5 cm wide)	1
Failure to exit a 30-cm-diameter circle (for 2 min)	1
Inability to walk straight	1
Loss of startle behavior	1
Loss of seeking behavior	1
Maximum total	10

One point is awarded for failure to perform a task

Wire grip tests were used to evaluate vestibular motor function. A 45-cm-long, 3-mm-diameter metal wire was suspended 45 cm above the ground with two vertical wooden sticks. Mice were placed on the bar midway between the supports and were observed for 60 s, and then the latency to fall was recorded and assessed.

### Open field test (OFT)

As previously reported [34], On day 14 after TBI, we used the open-field experiment to assess anxiety behavior in mice. The equipment used for the open-field experiment consisted of an organic 50 cm×50 cm×50 cm polyvinyl chloride box. The bottom was uniformly divided into 25 small compartments, and its interior and bottom were covered with a black opaque and non-reflective coating. A digital camera was installed 2 m above the plate. The entire interior of the field was visible, with the 9 center grids in the central area and the remaining grids in the peripheral area. A video tracking system (ANY-maze Stoelting USA) was used to monitor the behavior of the mice, and the entire measurement process lasted for 5 min. The overall time in the center area and the total distance during the recording were recorded and analyzed. The experiment time is between 10am and 3pm at a quiet environment.

### Morris water maze test (MWM)

Cognitive function was assessed using the Morris water maze test for 6 consecutive days. Briefly, the latency to find the platform was measured from day 15 to 19 post-CCI using the navigation test. The time spent in the correct quadrant was measured on day 20 post-CCI using the probe trial test [33]. The Morris water maze test was performed using a swimming pool (1.2 m diameter,40 cm height). A platform (8 cm diameter,20 cm height) was placed in one of four quadrants of the pool. The pool was filled with water (22 ± 1 °C) to a depth of 5 cm above the platform. Visual cues (i.e., black landmark) were placed on the wall of the water maze. The mice behavior was recorded using an camera that was placed above the pool, and ANYmaze 7.0 software system was used for recording and analysis. The data were exported to Microsoft Excel. The order of the animals was randomized within trials. The test consisted of 5 days of training (four trials, 60 s each). Memory was tested at day 6. For the test, the platform was removed, and the animals were allowed to swim in the pool for 60 s. The time to locate the platform was measured during all training days. Swimming speed was also recorded to assess motor performance. Data from the spatial learning test and the memory test were recorded and analyzed using a video-based tracking system (ANY-maze, Stoelting, USA).

### FITC-dextran intestinal permeability assay

Measurement of serum concentration of 4-kDa fluorescein isothiocyanate-dextran (FITC-dextran) represents for intestinal permeability. Seven days after TBI, mice were fasted for 14 h and gavage with 4-kDa FITC-dextran (Sigma-Aldrich, Madrid, Spain) at a dose of 60 mg per 100 g body weight in a volume of 0.2 ml. Four hours later, blood was collected by cardiac puncture and clotted for 30 min, followed by centrifugation at 6000 g for 90 s. Serum was diluted equally with PBS, and 100 µL dilutions were measured in a 96-well plate at 481 nm excitation and 524 nm emission. FITC-dextran was quantified by reference to standard curve measurements in the same plate [29].

### Gastrointestinal transit assessment

GI motility was assessed by radiographic methods 7 days after TBI. Specifically, animals were intragastrically gavaged with barium (0.2 ml, 2 g/ml). All mice were anesthetized with isoflurane (3% induction, 2% maintenance) and immobilized in the supine position temporally by fixing the limbs on the exam stage with adhesive tapes. Plain radiographs of the gastrointestinal tract were obtained using a digital radiographer (Siemens, Germany). To further reduce stress, mice were released immediately after each imaging session (immobilization lasted 1–2 min). Radiographs were taken at different times (1, 3, and 8 h after barium administration). Analysis of the radiographs was performed by a trained investigator blinded to the different groups [35]. Changes in GI motility were determined semiquantitatively from the images by assigning a composite value to the indicated regions of the GI tract, taking into account the following parameters: percentage of the GI region filled with contrast (0–4); intensity of the contrast (0–4); homogeneity of the contrast (0–2); and sharpness of the profile of the GI region (0–2). Each of these parameters was scored and a total score (0–12 points) was calculated. X-ray images were also analyzed morphometrically using ImageJ software (ImageJ 1.4, NIH, Bethesda, MD, USA).

### Microbiome analysis

Feces from each mouse were collected in autoclaved sterile vials on day 7 after TBI and stored at − 80 °C. Samples were shipped to Shanghai Majorbio Bio-Pharm Technology Co., Ltd. for analysis. Briefly, bacterial genomic DNA was extracted, and the bacterial V3-V4 region was amplified using barcode-indexed primers (338 F, ACTCCTACGGGAGGCAGCAG; 806 R, ACTCCTACGGGAGGCAGCAG). PCR products were sequenced using the Illumina MiSeq platform and analyzed on the Majorbio Cloud platform [36].

### Metabolite analysis

Serum from each mouse was collected in eppendorf tubes on day 7 after TBI and stored at -80 °C. Samples were sent to Shanghai Biotree Biomedical Technology Co., Ltd. for analysis. Targeted metabolomics analysis was performed by the ultra-performance liquid chromatography coupled with triple-quadrupole tandem mass spectrometry (UPLC-QqQ-MS/MS) and was provided by BIOTREE (Shanghai, China) [37].

### Enzyme-linked immunosorbent assay (ELISA)

The brain tissue of ipsilateral injured hemisphere and feces were homogenized with an appropriate amount of normal saline (saline: fecal/brain tissue = 9 ml:1 g), and the supernatant was removed by centrifugation at 3000 rpm for 10 min at 4℃. The amount of GUO in the supernatant and serum was measured by a mouse cyclic guanosine monophosphate (guanosine) ELISA kit (MEIKE Jiangsu Sumeike Biological Technology Co., Ltd) according to the manufacturer’s instructions [38]. The amount of GUO was normalized to protein content.

### CAT, SOD, ROS and GSSG measurements

Brain samples from the ipsilateral injured hemisphere were collected. The activities of CAT, SOD, ROS, GSSG levels and GSH/GSSG ratio were measured using commercial kits (Nanjing Jiancheng Bioengineering Institute, Nanjing, China) according to the manufacturer’s instructions [39, 40]. Protein concentration was measured by the BCA kit method [41].

### Western blot analysis

Western blot analysis was performed as previously described [42]. Briefly, brain tissue including the injury site (approximately 5 mm length ×5 mm width×3 mm height), was collected for total protein extraction using RIPA lysis buffer and protease and phosphatase inhibitors. The sample proteins (20 µg/lane) were separated by sodium dodecyl sulfate–polyacrylamide gel electrophoresis (SDS‒PAGE) (Invitrogen) and transferred onto polyvinylidene fluoride (PVDF) membranes (Millipore, Boston, MA, USA). Membranes were blocked with 5% nonfat milk for 1 h at room temperature and then incubated overnight at 4 °C with primary antibodies, including rabbit monoclonal anti-β-actin (1:5000, ab213262, Abcam), rabbit polyclonal anti-ZO-1 (1:1000, 21773-1-AP, Proteintech), rabbit polyclonal anti-Occludin (1:1000, 27260-1-AP, Proteintech), rabbit monoclonal anti-Bcl-2 (1:1000, ab182858, Abcam), rabbit monoclonal anti-Bax (1:1000, ab182733, Abcam), rabbit polyclonal anti-p-Akt (1:1000, 28731-1-AP, Proteintech), rabbit polyclonal anti-Akt (1:1000, 10176-2-AP, Proteintech), rabbit polyclonal anti-p-PI3K (1:1000, #4228, Cell Signaling), and rabbit polyclonal anti-PI3K (1:1000, 60225-1-lg, Proteintech). After being washed with Tris-buffered saline/Tween-20, membranes were incubated for 1 h at room temperature with horseradish peroxidase-conjugated AffiniPure goat anti-rabbit IgG (1:5000; SA00001-2, RRID: AB_2722564, Proteintech). Enhanced chemiluminescence was used to detect proteins in the membranes(ECL Plus, Millipore), and proteins were quantified using the Fusion system (Fusion-FX7 Spectra, Vilber, France). All raw Western blot bands are shown in Supplemental Figures.

### Immunofluorescence staining

As previously reported [42], the mice were euthanized under deep anesthesia with phenobarbital sodium (40 mg/kg, i.p.) and then perfused with phosphate buffer saline (PBS) and 4% paraformaldehyde (PFA) for fixation. The collected brains were postfixed overnight at 4 °C in 4% PFA and then cryoprotected in graded sucrose (20% and 30%). Next, the brains were embedded in optimal cutting temperature compound and cut into 20-µm coronal sections. These tissues were stained using a slide-mounted method. After washing with PBS and PBS + 0.4% Triton X-100, the brain sections were blocked with 10% goat serum for 1 h at 37 °C, incubated with primary antibodies overnight at 4 °C and washed three times with PBS. Then, the sections were incubated with secondary antibodies (1:400, Beyotime Institute of Biotechnology, Shanghai, China) conjugated to Alexa Fluor 488/594 for 1 h at room temperature. Cell nuclei were stained with 4′,6-diamidino-2-phenylindole (DAPI, 1:1, Solarbio). The primary antibodies included rabbit polyclonal anti-ZO-1 (1:1000, 21773-1-AP, Proteintech), rabbit polyclonal anti-Occludin (1:1000, 27260-1-AP, Proteintech), mouse monoclonal anti-CD31 (1:50, GTX20218, Genetex), and mouse monoclonal anti-NeuN (1:100, 66836-1-Ig, Proteintech). For each sample, 3–4 corresponding sections at intervals of 300 μm were collected. For each group, sections from 4 mice were used for analysis. Each measurement was expressed as the average of all section measurements per mouse. All sections were collected where the lesions were located. The regions of interest (ROIs) where the images were captured are shown in the black box of Fig. 8D. All images were captured using a Leica DM4 B fluorescence microscope (Leica, DM4 B, Wetzlar, Hesse, Germany) with a 10× eyepiece and a 20× objective and processed using ImageJ. We set the same exposure time when quantitative analysis was involved in each experiment. The relative immunofluorescence intensity of Occludin was calculated by the percentage of immunofluorescence intensity of Occludin relative to immunofluorescence intensity of CD-31 with ImageJ. Additionally, the relative immunofluorescence intensity of Occludin/ZO-1 in the intestinal mucosa was also calculated by the percentage of immunofluorescence intensity of Occludin/ZO-1 relative to immunofluorescence intensity of DAPI with ImageJ software. All counts were obtained in a blinded fashion.

TUNEL assays were performed using a one-step TUNEL kit (C1090, Beyotime Institute of Biotechnology, Shanghai, China) according to the manufacturer’s instructions. These tissues were stained using a slide-mounted method just as above. The immune-positive cell numbers were calculated with ImageJ, and presented as the mean number of cells per high power field (HPF) for single staining or presented as the percentage of the number of cells for the main marker to the number of cells for the secondary marker per HPF for double-staining.

### BBB permeability assays

To measure BBB permeability, 2% Evans blue (EB, 4 mL/kg) in sterile saline was injected through the tail vein 1 h before the animals were sacrificed. The mice were transcardially perfused with saline, and their brains were dissected and weighed. The samples were then homogenized in PBS, sonicated for 2 min, and centrifuged at 15,000 rpm for 5 min at 4 °C, and the supernatant was then collected in aliquots. Next, 500 µL of 50% trichloroacetic acid was added to each 500 µL of supernatant and incubated overnight at 4 °C. Finally, these samples were centrifuged at 15,000 rpm for 30 min at 4 °C, the supernatant was collected and detected with a spectrometer at 610 nm and quantified using a standard curve that was normalized to tissue weight (µg/g). Then, to assess the fluorescence intensity, the brains were removed in preparation for coronal brain sectioning. Brains for Evans blue fluorescence were removed and fixed in 4% paraformaldehyde at 4 °C for 24 h and prepared for coronal brain sectioning (20 μm). Red auto-fluorescence of Evans blue was observed on the Leica DM4 B fluorescence microscope with a 10× eyepiece and a 20× objective and processed using ImageJ [43]. The mean red autofluorescence of EB was evaluated by 2 observers blinded to the groups.

### Transmission electron microscopy

After anesthetization, the mice were transcardially perfused with ice-cold saline followed by 4% PFA and 2.5% glutaraldehyde with 0.1 mol/L PBS buffer. The pericontusional cortex tissues were microdissected into 1 mm^3^ specimens. Then, these samples were immersion-fixed in 2% glutaraldehyde for 24 h. After washing with PBS, they were fixed in 1% osmium tetroxide in 0.1 M PBS for 45 min. The specimens were dehydrated in increasing concentrations of acetone and embedded in Aralditeresin. Ultrathin 60-nm-thick sections were cut using a diamond knife on a Leica UCT ultramicrotome (Diatome, Wetzlar, Germany). The prepared ultrathin sections were mounted on a copper grid, stained with uranium acetate and lead citrate, and observed with a JEM-1400Plus TEM (H-7500, Hitachition) as previously described [44].

### Statistical analysis

All results are presented as the means ± standard deviations (SDs). Before analysis, the Shapiro–Wilk test was used to test the normality of the variables. Two-way repeated-measures ANOVA with Tukey’s post hoc multiple-comparisons test was used to analyze continuously measured data. One-way analysis of variance (ANOVA) was used to compare the means of different groups followed by a Tukey post hoc multiple-comparisons test. All statistical analyses were performed using GraphPad Prism software (version 9.5.1, CA, USA). In the 16 S rRNA gene sequencing analysis, alpha diversity indices (Ace, Chao and sobs even indices) were calculated in QIIME (version 1.7.0) and examined using the Kruskal‒Wallis H-test in SPSS 21.0. Principal coordinate analysis (PCoA) based on Bray‒Curtis similarities. The Kruskal‒Wallis H-test with false discovery rate (FDR), multiple comparisons correction, and Tukey’s post hoc comparisons test were calculated at the phylum, family, and genus levels among each group, and corrected *p* < 0.05 was considered statistically significant [14]. For metabolomics analysis, orthogonal partial least-squares discriminant analysis (OPLS-DA), a multivariate statistical analysis model, was conducted with SIMCA16.1 software (Umetrics, Umeå, Sweden). OPLS-DA models were validated based on the multiple correlation coefficient (R2) and cross-validated Q2 in cross-validation and permutation tests by applying 200 iterations. A Q2 value of more than 0.3 showed that the model was stable and reliable. The permutation tests are deemed valid when all Q2 and R2 values show a gradual decline. The variable importance in the projection (VIP) value of each variable in the OPLS-DA model was calculated to indicate its contribution to the classification. The model validity was evaluated by 7-fold cross-validation, and a permutation test (*n* = 200) VIP > 1 and *P* < 0.05 were used as screening thresholds for differentially abundant metabolites [37]. Statistical significance was defined as *p* < 0.05.

## Results

### Time course of *P. Copri* abundance after TBI

**Fig. 2:**
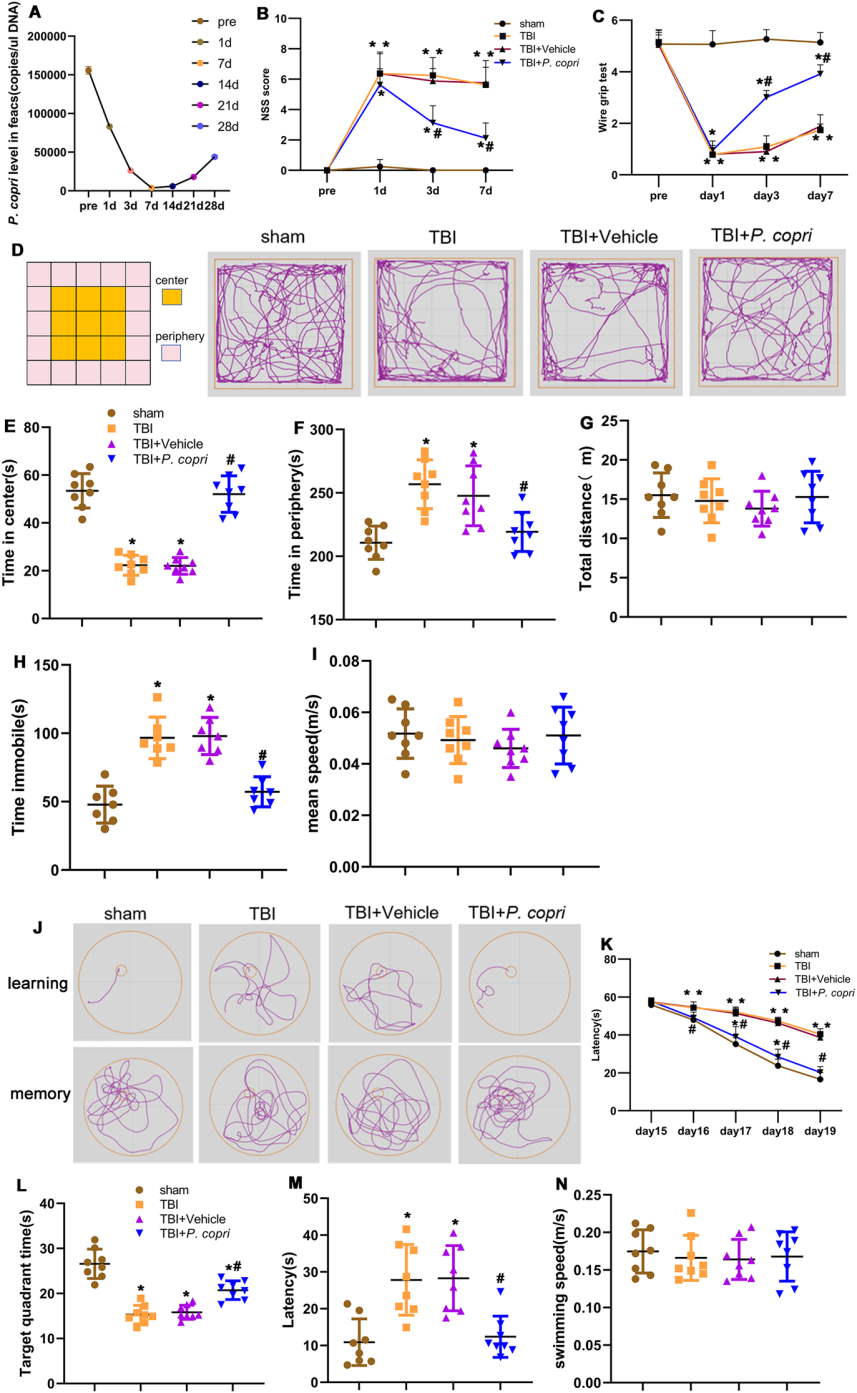
*P. copri* treatment improved neurological function in TBI mice. **A**. Time course of *P. copri* abundance after TBI by qPCR. B-C. Quantitative analysis of short-term neurological function by NSS scores (**B**) and wire grip scores (**C**). *, *p* < 0.05 vs. sham; #, *p* < 0.05 vs. TBI. *n* = 8 per group. **D**. Representative images of the movement path of mice in the open field test 14 days after TBI. E-I. Quantitative analysis of central time (**E**), periphery time (**F**), total distance(**G**), time immobile (**H**) and mean speed (**I**) travelled by mice in the open field test. *, *p* < 0.05 vs. Sham; #, *p* < 0.05 vs. TBI, *n* = 8 per group. **J**. Representative images of the swim path of mice in the Morris water maze. K-N. Quantitative analysis of the latency in the learning test (**K**), the time in the target quadrant (**L**), the latency (**M**) and the average swimming speed (**N**) in the probe trial. *, *p* < 0.05 vs. Sham; #, *p* < 0.05 vs. TBI, *n* = 8 per group. Two-way repeated-measures ANOVA with Tukey’s post hoc multiple-comparisons test was used to analyze continuously measured data. One-way analysis of variance (ANOVA) was used to compare means of different groups followed by a Tukey post hoc multiple-comparisons test

qPCR was used to assess the abundance of *P. copri* in the feces of mice at the following timepoint: before TBI, 1 d, 3 d, 7 d, 14 d, 21 d, and 28 d post-TBI. After TBI, we found that the abundance of *P. copri* decreased gradually over time; its abundance was lowest at 7 d and then increased gradually at 14 d, 21 d, and 28 d. However, its abundance at 28d post-TBI still did not reach the level before TBI (Fig. 2A).

### *P. Copri* treatment improved neurological function in TBI mice

The NSS score and wire grip scores were applied to evaluate the effect of *P. copri* transplantation on neurological deficits. The injury groups, which included the TBI group, TBI + Vehicle group and TBI + *P. copri* group, had significantly higher NSS scores than the sham group on day 1, day 3, and day 7 after TBI. The TBI + *P. copri* group had lower NSS scores than the TBI group and TBI + Vehicle group on day 3 and 7 after TBI, while there were no significant differences between the NSS scores of the TBI group and TBI + Vehicle group (Fig. 2B). The injury groups, which included the TBI, TBI + Vehicle, and TBI + *P. copri* groups, had significantly lower wire grip scores than the sham group on day 1, day 3, and day 7 after TBI. The TBI + *P. copri* group had higher wire grip scores than the TBI group and TBI + Vehicle group on day 3 and day 7 after TBI, while there were no significant differences between TBI group and TBI + Vehicle groups (Fig. 2C).

Next, the open field test (OFT) was used to assess anxiety related behavior 14 days after TBI. Our results showed that TBI resulted in less time spent in the center, more time spent in the periphery and immobile in the OFT than the sham group, indicating that mice exhibited anxiety related behavior after TBI. However, *P. copri* transplantation reversed this effect compared to the TBI group and TBI + Vehicle group. In addition, there was no significant difference between the TBI group and the TBI + Vehicle group (Fig. 2D, E, F, H). There was no significant difference among the four groups in the total distance traveled and mean speed in the OFT (Fig. 2G, I).

In addition, the Morris water maze was performed to evaluate spatial learning and memory on days 15 to 20 after TBI. In the learning stage, mice in the TBI, TBI + Vehicle and TBI + *P. copri* groups took more time to find the correct target than those in the sham group. There was no significant difference between the TBI and the TBI + Vehicle groups. However, *P. copri* treatment significantly decreased the latency in the learning test compared with the TBI group and the TBI + Vehicle group (Fig. 2J and K). In the probe trial, mice in the TBI and TBI + Vehicle group took more time to enter the platform for the first time than those in the sham group, but *P. copri* treatment decreased the latency (Fig. 2M). Meanwhile, TBI resulted in less time spent in the correct target quadrant than that in the sham group, but *P. copri* treatment increased the time spent in the target quadrant when compared to the TBI group and TBI + Vehicle group (Fig. 2L). There was no significant difference in swimming speed among the groups of mice (Fig. 2N).

### *P. Copri* treatment improved intestinal permeability and motility in TBI mice

**Fig. 3:**
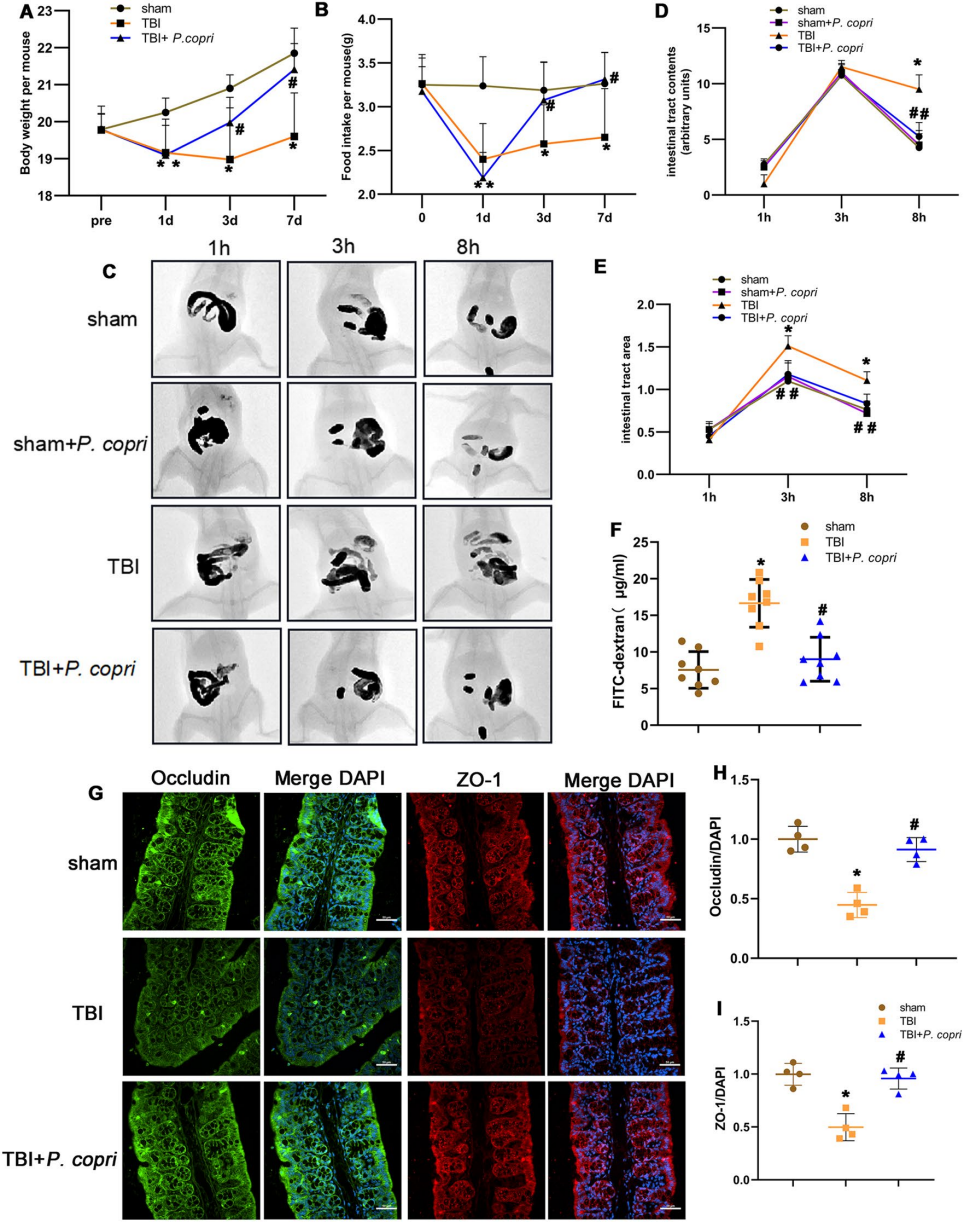
*P. copri* treatment improved body weight, food intake, intestinal permeability and intestinal motility of TBI mice. **A**-**B**. Changes of body weight (**A**) and food intake (**B**) over time. *, *p* < 0.05 vs. sham; #, *p* < 0.05 vs. TBI. *n* = 8 per group. **C**-**E**. Effect of *P. copri* on gastrointestinal motility. Representative images after administration of barium (**C**), the filling of the intestinal tract was measured by radiological methods (**D**), the size of the intestinal tract was determined using ImageJ (**E**). *, *p* < 0.05 vs. Sham; #, *p* < 0.05 vs. TBI. *n* = 4 per group. **F**. Intestinal permeability by measuring FITC intensity in serum after oral gavage of FITC-dextran. *, *p* < 0.05 vs. sham; #, *p* < 0.05 vs. TBI. *n* = 8 per group. G-I. Effect of *P. copri* treatment on tight junction proteins expression. Representative images of immunofluorescence staining for Occludin (green) and ZO-1 (red), nuclei were stained with DAPI (blue) (**G**). Scale bar = 100 μm. Quantitative analysis of relative fluorescence intensity of Occludin (**H**) and ZO-1 (**I**) in different groups. *, *p* < 0.05 vs. Sham; #, *p* < 0.05 vs. TBI, *n* = 4 per group. Two-way repeated-measures ANOVA with Tukey’s post hoc multiple-comparisons test was used to analyze continuously measured data. One-way ANOVA was used to compare means of different groups followed by a Tukey post hoc multiple-comparisons test

*P. copri* transplantation has been reported to be safe in mice [30]. In our study, body weights and food intake were monitored and compared at the indicated time points. The body weights of the mice were greatly reduced in the TBI group and increased gradually after *P. copri* treatment (Fig. 3A). The food intakes of mice were also greatly reduced in the TBI group and increased gradually after P. copri treatment (Fig. 3B).

Altered intestinal barrier integrity and subsequent gastrointestinal dysfunction have been postulated as pathophysiological events in TBI [45, 46]. GI transit, as an overall measure of GI motility, was assessed in mice by using barium gavage followed by X-ray imaging. Representative images from different groups at different time points are shown in Fig. 3C (1, 3, and 8 h after gavage). Overall, TBI mice had a higher level of barium filling in the GI tract, which indicates a slower GI transit. *P. copri* treatment accelerated GI transit in mice after TBI. With respect to gut tract filling, GI motility was significantly delayed after TBI at 8 h after barium gavage, which was improved in TBI + *P. copri* mice. Meanwhile, there was no significant difference between the sham group and the sham + *P. copri* group (Fig. 3D-E). Taken together, *P. copri* treatment may have contributed to the faster GI transit following TBI.

To test whether *P. copri* treatment had any effect on intestinal barrier permeability after TBI, the mice were gavaged with FITC-labeled dextran (4 kDa) on day 7 and blood FITC levels were measured. Intestinal permeability was more severely disrupted in the TBI group than in the TBI + *P. copri* group (Fig. 3F). Intestinal tight junctions (TJs) have been shown to be associated with intestinal barrier integrity [47]. Therefore, in our study, we examined the expression and distribution of TJ proteins in the colon. In the TBI group, the relative immunofluorescence intensity of Occludin and ZO-1 in the intestinal mucosa were reduced, and *P. copri* treatment increased the relative immunofluorescence intensity of Occludin and ZO-1 (Fig. 3G-I). The results demonstrated that *P. copri* treatment led to an upregulation of tight junction proteins expression and facilitated the maintenance of intestinal barrier integrity after TBI.

### *P. Copri* treatment reshaped the gut microbiota of TBI mice

**Fig. 4:**
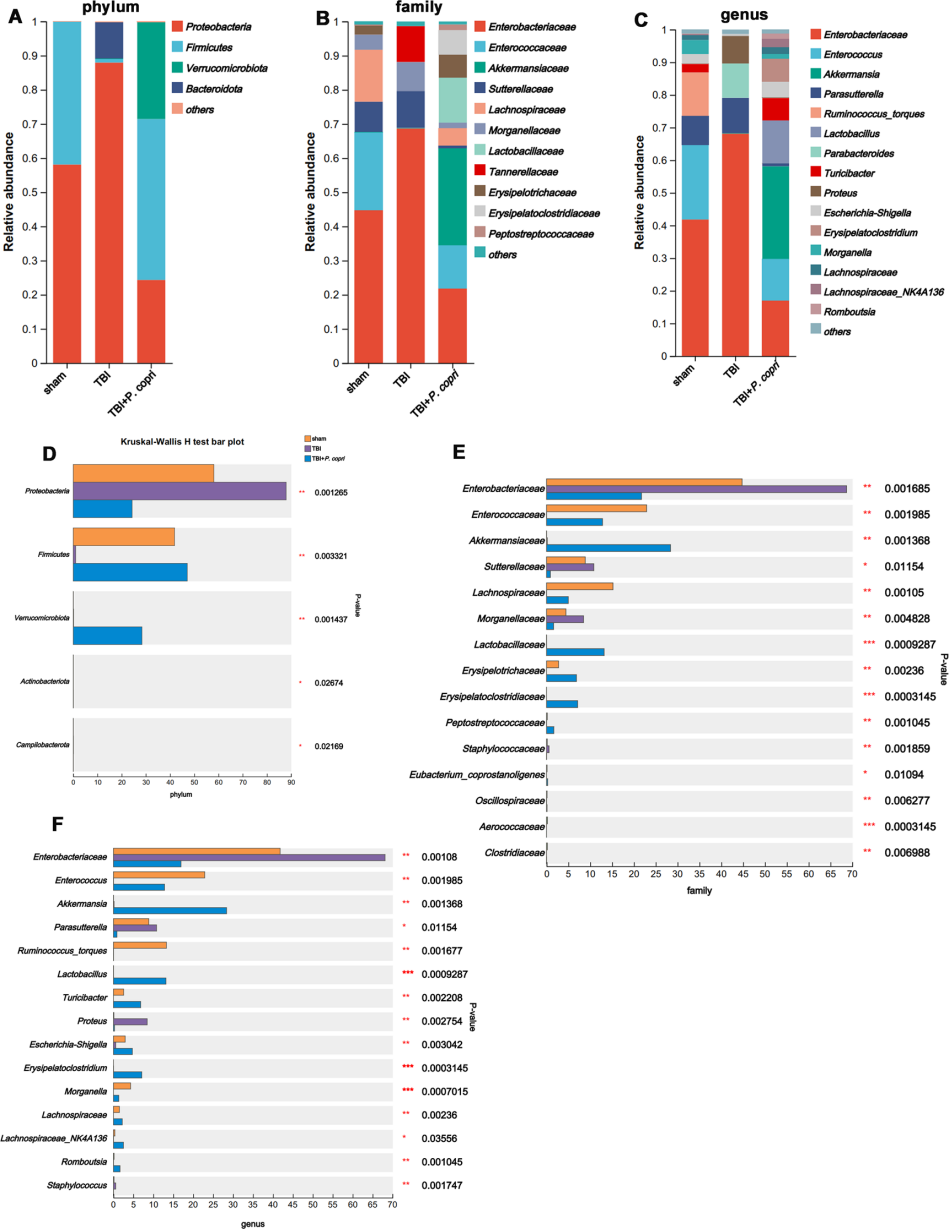
*P. copri* treatment reshaped the gut microbiota of TBI mice. **A**-**C**. Bar plot analysis of gut microbiota relative abundance of bacterial phylum (**A**), family (**B**) and genus (**C**) in the sham, TBI, TBI + *P. copri* groups. Different colours represent different phyla, family and genus. **D**-**F**. Significant differences in the abundance of phyla (**D**) in each group, and significant differences in the families (**E**) and genus (**F**) with high abundance (top 15) (*n* = 6 per group). Data are presented as mean ± SD. *, *p* < 0:05. **, *p* < 0:01. ***, *p* < 0:001. The Kruskal‒Wallis H-test with false discovery rate (FDR), multiple comparisons correction and Tukey’s post hoc comparisons test were calculated at the phylum, family, and genus levels among each group

To test whether *P. copri* treatment modulated the gut microbiota, we performed 16 S rDNA (V3 + V4 regions) gene sequencing to analyze the bacterial taxonomic composition following microbial therapy in TBI mice. To measure the degree of similarity between microbial communities, β diversity was further evaluated by using Bray‒Curtis principal coordinate analysis (PCoA), which showed apparent clustering in different groups (Suppl Fig. 1A-C). The α-diversity was measured by the Ace, Chao and Sobs even indices. Results from the analysis showed that the α-diversity index was significantly different among the three groups (Suppl Fig. 1D-F). Community bar plot analysis, at the phylum level, based on taxonomic analysis, revealed 4 different phyla when compared with the sham and TBI + *P. copri* groups, such as *Proteobacteria, Firmicutes, Verrucomicrobiota*, and *Bacteroidetes* (Fig. 4A). Further analysis revealed that the relative abundances of the phyla *Firmicutes and Verrucomicrobia* were lower and the relative abundance of *Proteobacteria* was increased in the TBI group compared to the sham or TBI + *P. copri* groups (Fig. 4D). At the family level, the TBI group had 11 different families compared with the sham and TBI + *P. copri* groups (Fig. 4B), and species difference analysis showed that *Enterobacteriaceae*, *Sutterellaceae*, *Morganellaceae* and *Staphylococcaceae* were higher in the TBI group than in the sham and TBI + *P.copri* groups, and *P. copri* treatment increased the relative abundance of *Enterococcus*, *Akkemansia*, *Lachnospiraceae*, *Lactobacillus*, *Erysipelotrichaceae*,

*Eryssipelatoclostridiaceae* and *Peptostreptococcaceae* (Fig. 4E). At the genus level, the TBI group had 15 different bacteria when compared with the sham and TBI + *P. copri* groups (Fig. 4C). Species difference analysis showed that in the TBI group induced alterations of 3 genera with high relative abundance, namely, *Enterobacteriaceae, Parasulterella and Proteus*, and in the TBI + *P. copri* group, the relative abundances of *Enterococcus*, *Akkemansia*, *Lactobacillus*, *Turicibacter*, *Escherichia-Shigella*, *Eryssipelatoclostridium*, *Morganella*, *Lachnospiraceae*, *Lachnospiraceae-NK4A136* and *Romboutsia* were increased compared to those in the TBI group (Fig. 4F).

### *P. Copri* treatment caused changes in serum metabolomics in TBI mice

**Fig. 5:**
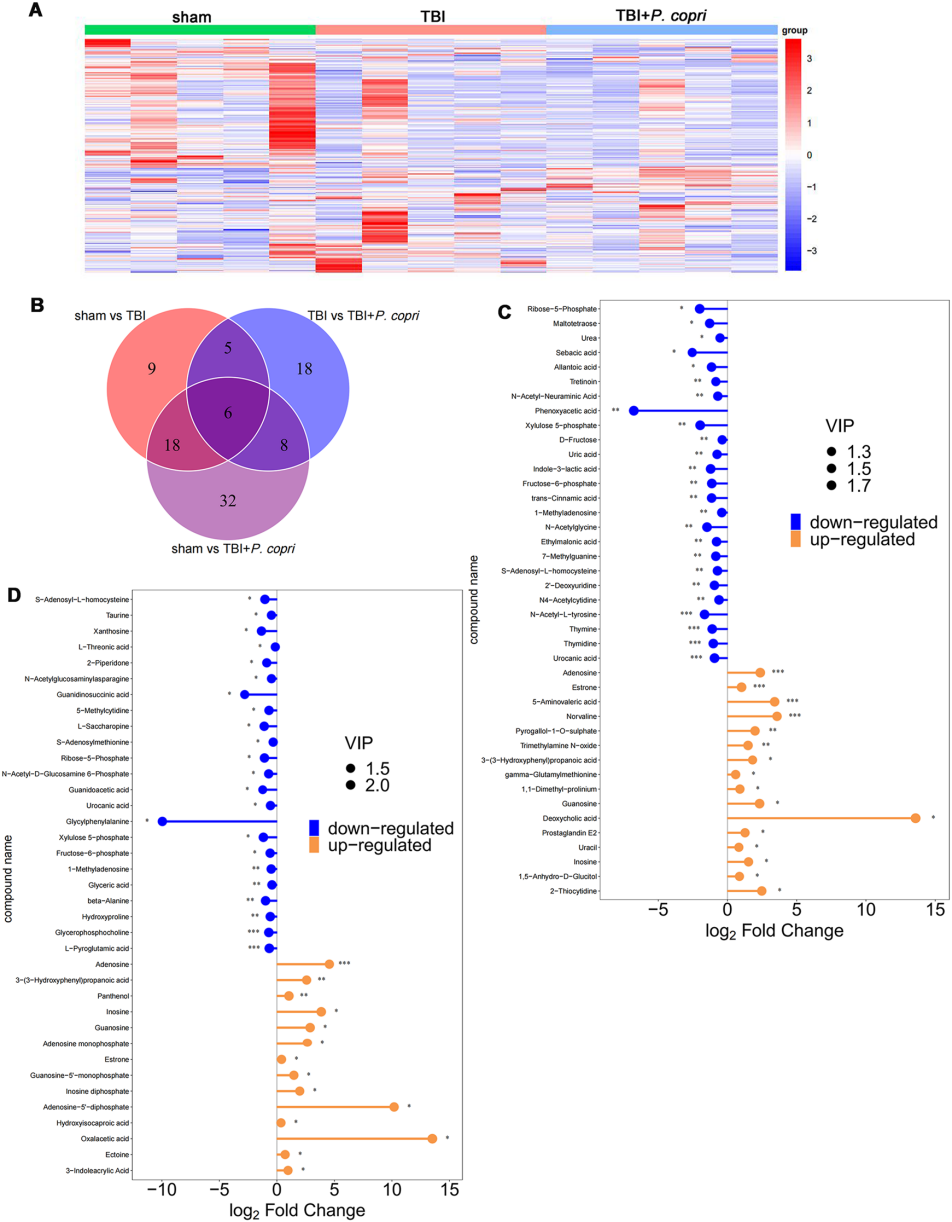
*P. copri* treatment caused the changes in serum metabolomics in TBI mice. **A**. Hierarchical clustering analysis of serum metabolite biomarkers. The abscissa is the clustering of samples and the ordinate is the clustering of differential metabolites. The coloured blocks in different positions represent the content of the metabolites in the corresponding parts. Red means that the substance is highly expressed in the group where it is located, while blue means that the sense is poorly represented in the group where it is located. **B**. Venn diagram of differential metabolites in serum. **C**-**D**. Matchstick analysis of serum metabolite biomarkers. TBI + *P. copri* group vs. sham group (**C**), TBI + *P. copri* group vs. TBI group (**D**). Red nodes represent significantly upregulated metabolites (top 25), blue nodes represent significantly downregulated metabolites (top 25) (VIP > 1 and *P* < 0.05)

The results of behavioral testing showed that vehicle treatment does not impact neurological behaviors after TBI, therefore we choose the sham group, TBI group and TBI + *P. copri* groups for in-depth metabolomics analysis. To profile differential metabolism, OPLS-DA was performed using these three groups, which were compared in pairs and possessed satisfactory fit. The results showed a clear separation in the two groups (Suppl Fig. 2A-C). Permutation tests with 200 iterations were performed, which also validated these models. In the serum, 38 metabolites were found to be differentially expressed between the sham group and the TBI group, 37 metabolites were differentially expressed between the TBI group and the TBI + *P. copri* group, 64 metabolites were differentially expressed between the sham group and the TBI + *P. copri* group (Fig. 5A and B). Combining the matchstick analysis of the serum metabolites suggested that guanosine (GUO) was elevated simultaneously in serum of the TBI + *P. copri* group when compared to the sham group and the TBI group (VIP > 1 and *p* < 0.05, Fig. 5C and D).

### *P. Copri* treatment increased the level of GUO in the feces, serum and brain tissue of TBI mice

**Fig. 6:**
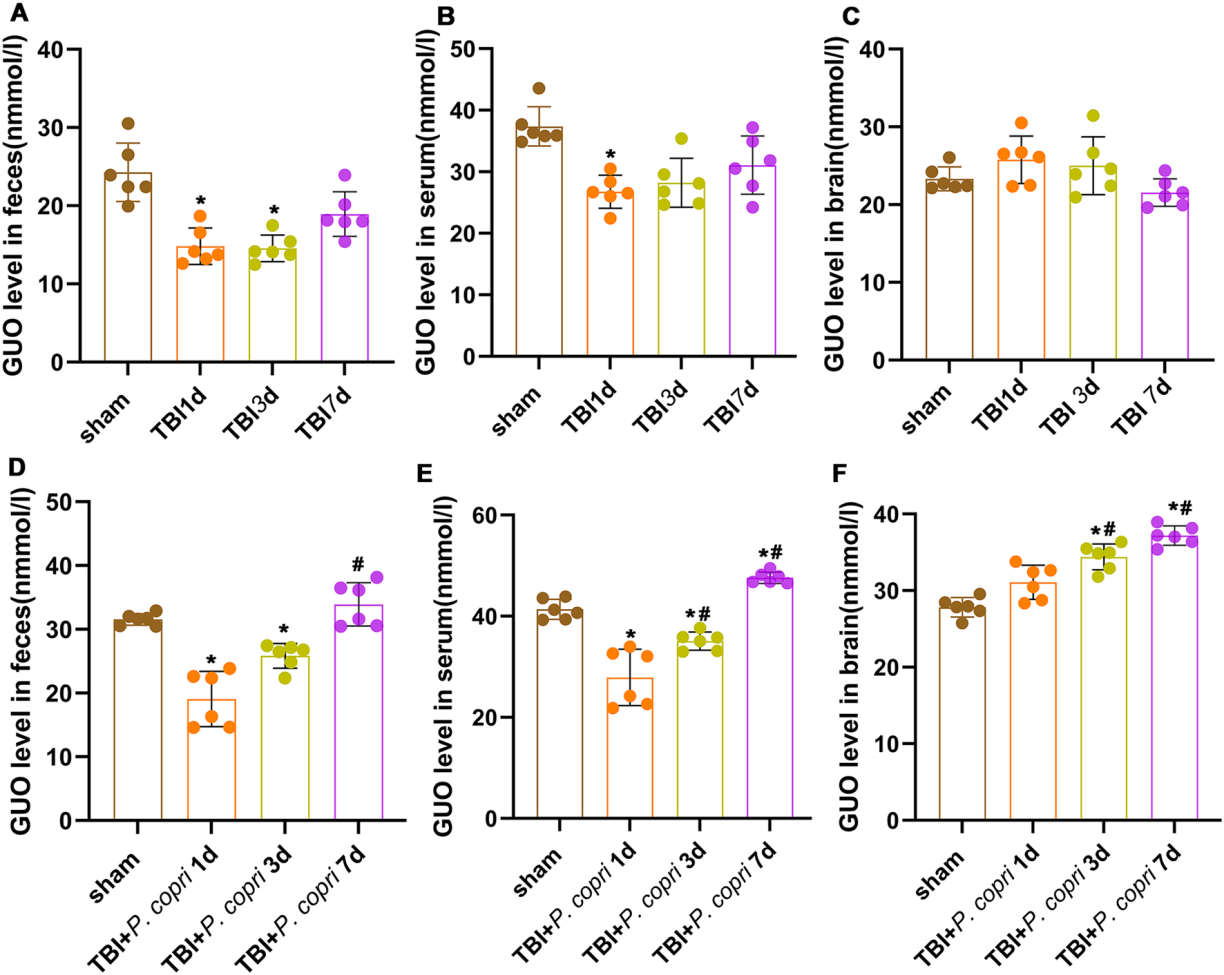
*P. copri* treatment increased the presence of GUO in the feces, serum and brain tissue of TBI mice. **A**-**C**. The changes of GUO level in feces (**A**), serum (**B**) and brain (**C**) after TBI were detected by ELISA. *, *p* < 0.05 vs. sham; #, *p* < 0.05 vs. day 1. *n* = 6 per group. D-F. Changes in GUO level in feces (**D**), serum (**E**) and brain (**F**) after

In metabolomics analysis, GUO was one of the most significantly upregulated metabolites after *P. copri* treatment, thereafter ELISA analysis of GUO was subsequently performed. The level of fecal GUO after TBI at the 1st day and 3rd day were significantly decreased, and there is no significant difference at day 7 compared with sham group (Fig. 6A). The level of serum GUO after TBI at the 1st day was significantly decreased, and there is no significant difference at day 3 and day 7 compared with sham group (Fig. 6B). In the brain tissue, the change between each group did not reach statistical significance after TBI (Fig. 6C). After *P. copri* treatment, compared to the sham group, the level of fecal GUO was significantly decreased on the 1st day and 3rd day. Compared with the 1st day, the level of GUO increased gradually at 7th day (Fig. 6D). The level of serum GUO was significantly decreased on the 1st day and 3rd day but increased at 7th day compared to the sham group. Compared with the 1st day, the level of serum GUO increased gradually at 3rd and 7th day (Fig. 6E). The level of brain GUO after.

*P. copri* treatment at 3rd day and 7th day was significantly higher than that in the sham group as well as the 1st day group (Fig. 6F).

*P. copri* treatment. *, *p* < 0.05 vs. sham; #, *p* < 0.05 vs. day 1. *n* = 6 per group. One-way ANOVA was used to compare the means of different groups followed by a Tukey post hoc multiple-comparisons test.

### *P. copri* treatment attenuated oxidative stress after TBI

**Fig. 7:**
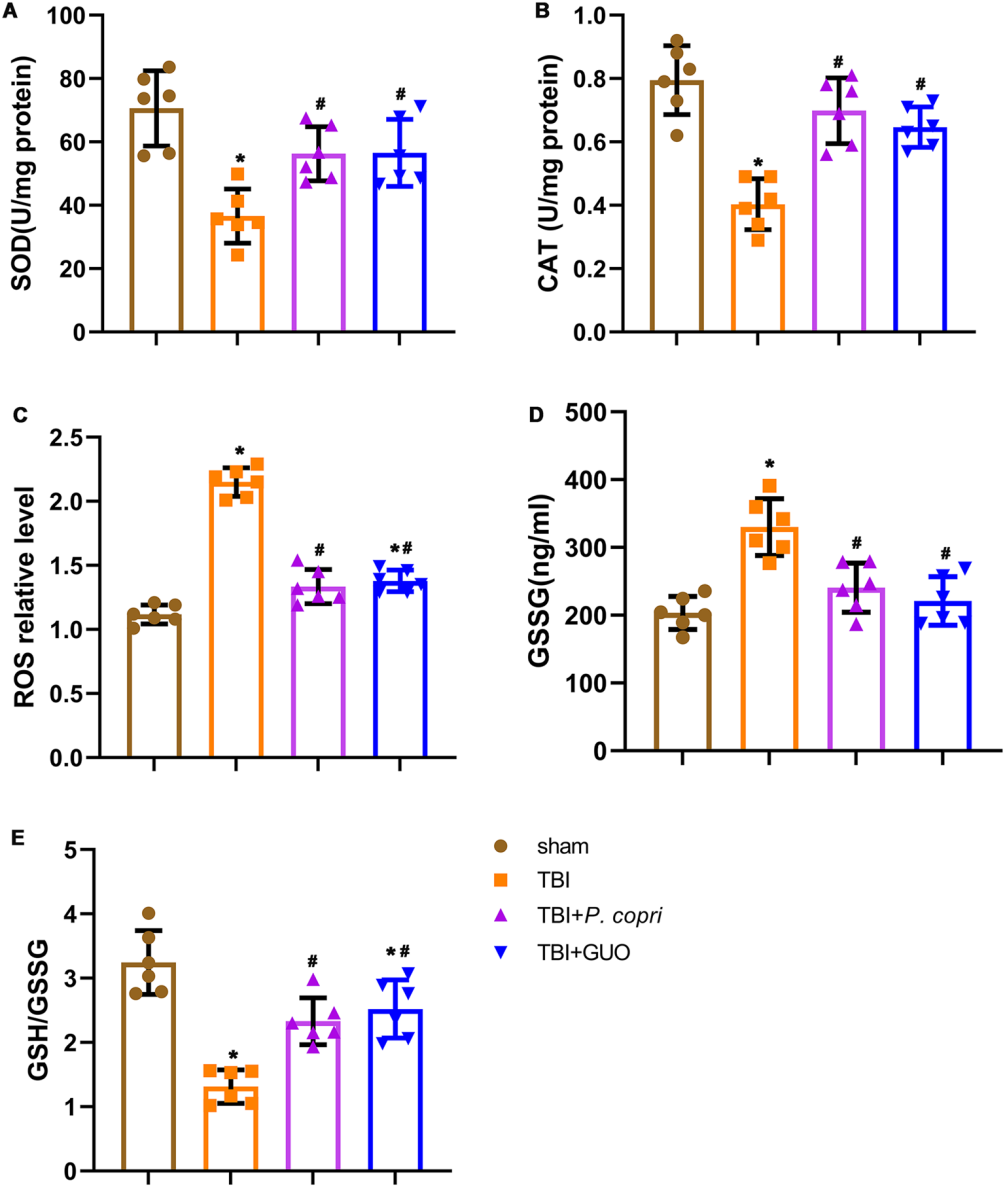
*P. copri* treatment attenuated oxidative stress after TBI. **A**-**E**. The changes of oxidative stress markers in the brain lesion area after TBI were detected by spectrophotometer. **A**. SOD activity. **B**. CAT activity. **C**. ROS level. **D**. GSSG level. **E**. GSH/GSSG ratio. *, *p* < 0.05 vs. sham; #, *p* < 0.05 vs. TBI. *n* = 6 per group. One-way ANOVA was used to compare the means of different groups followed by a Tukey post hoc multiple-comparisons test

The TBI group showed a significant decrease in CAT and SOD activities compared to the sham group. The TBI group showed higher levels of ROS and GSSG, along with a lower GSH/GSSG ratio than the sham group. After treatment with *P. copri* transplantation or GUO intake, both the TBI + *P. copri* group and the TBI + GUO group showed significant increases in CAT and SOD activities compared to the TBI group (Fig. 7A and B). In addition, the TBI + *P. copri* group and the TBI + GUO group had significantly reduced ROS and GSSG levels and an increased GSH/GSSG ratio compared to the TBI group (Fig. 7C-E). All of these results indicated that *P. copri* or GUO treatment can effectively reduce oxidative stress after TBI.

### *P. Copri* treatment attenuated BBB disruption after TBI

**Fig. 8:**
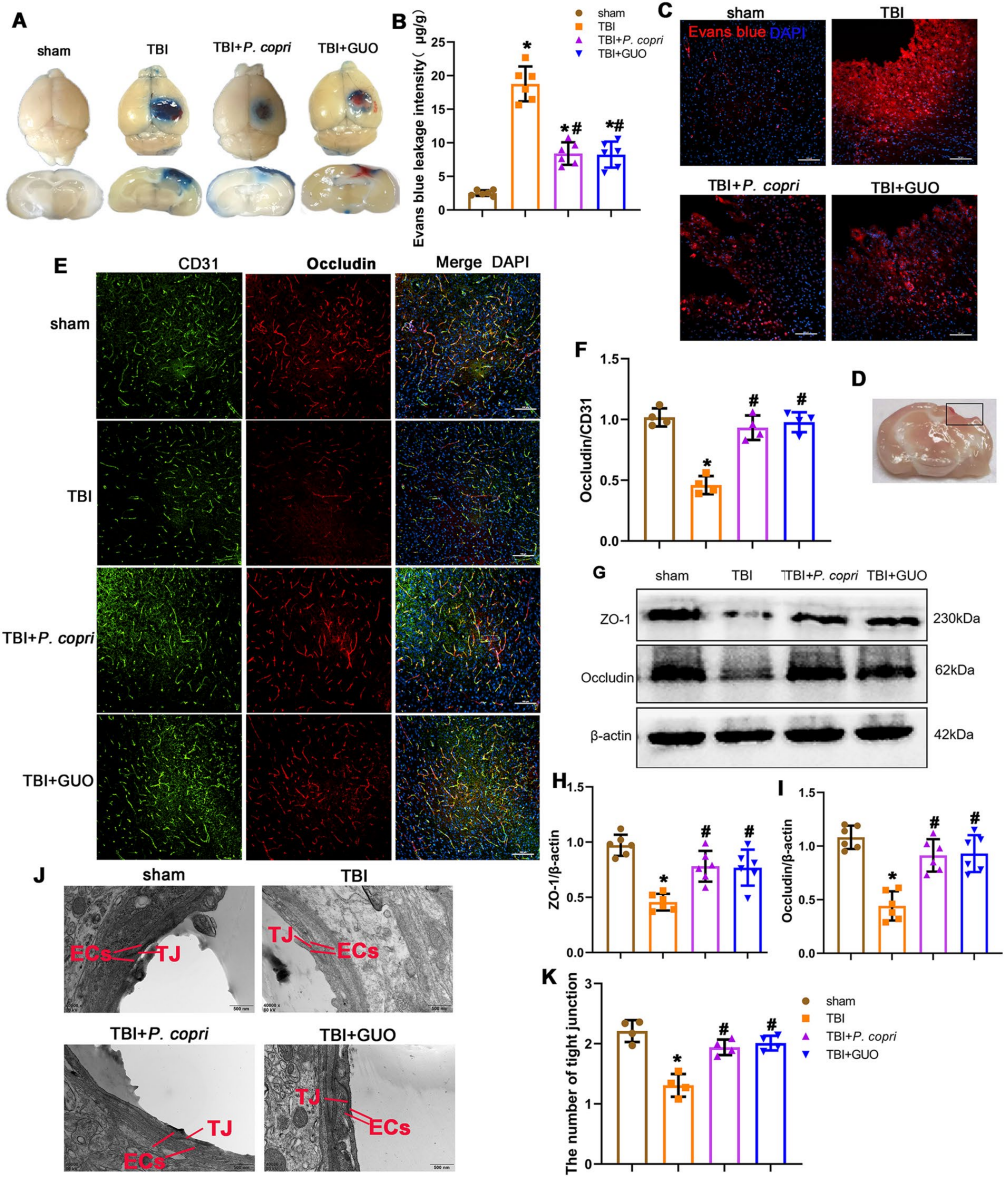
*P. copri* treatment attenuated BBB disruption after TBI. **A**. Representative horizontal and coronal images of brain slices after EB injection. **B**. Quantitative analysis of EB leakage intensity. *p, < 0.05 vs. sham; #, *p* < 0.05 vs. TBI, *n* = 6 per group. **C**. Red fluorescence of EB was observed by fluorescence microscopy in different groups. Cell nuclei were stained with DAPI (blue). Scale bar = 100 μm. *n* = 4 per group. **D**. Brain sample with a schematic illustration showing the area (indicated by the black box) that was used for the immunofluorescence analysis. **E**. Representative images of double immunofluorescence staining for Occludin and CD31, nuclei were stained with DAPI (blue). Scale bar = 100 μm. **F**. Quantitative analysis of relative Occludin fluorescence intensity in different groups. *, *p* < 0.05 vs. sham; #, *p* < 0.05 vs. TBI, *n* = 4 per group. **G**. Representative Western blot bands of ZO-1, Occludin and β-actin at the lesion sites after TBI. H-I. Quantitative analysis of relative ZO-1 (**H**), Occludin (**I**) density. *, *p* < 0.05 vs. sham; #, *p* < 0.05 vs. TBI, *n* = 6 per group. **J**. Transmission electron microscopy showed the ultrastructure of tight junctions. Scale bar = 500 nm. EC: Endothelial cells. TJ: Tight junction. **K**. Quantitative data show a decreased number of organized tight junctions. *, *p* < 0.05 vs. sham; #, *p* < 0.05 vs. TBI, *n* = 4 per group. One-way ANOVA was used to compare the means of different groups followed by a Tukey post hoc multiple-comparisons test

Evans blue assays indicated that TBI caused more Evans blue leakage than the sham group, and *P. copri* and GUO visually mitigated EB dye leakage after Evans blue injection (Fig. 8A-C). Meanwhile, double-immunofluorescence staining showed that Occludin was decreased significantly in the TBI group compared to the sham group, and treatment with *P. copri* and GUO attenuated this damage compared with the TBI group (Fig. 8E and F). Similarly, Western blot assays revealed that the expression of BBB-associated proteins (ZO-1, Occludin) was decreased significantly after TBI compared to sham group. By contrast, both *P. copri* treatment and GUO treatment showed an increase level compared to that in the TBI group (Fig. 8G-I).

Furthermore, we investigated the ultrastructure of the tight junctions by transmission electron microscopy analysis. In the sham group, we observed smooth and continuous TJ structures between endothelial cells, and the basement membrane was intact. After TBI, the microvilli of endothelial cells protruded, the continuity was interrupted, the density of intercellular TJ structures was reduced, and the opening is increased to form cracks. After treatment with *P. copri* or GUO, the above manifestations were improved: the opening of intercellular TJ structures was reduced, and the basement membrane was relatively intact (Fig. 8J). A scoring system was used to quantitatively determine the number of intact tight junctions, which were rated as follows: perfect tight junctions worth 3 points; patches of blurriness worth 2 points; totally blurred worth 1 point. In the TBI group, the number of intact tight junctions was significantly lower than that in the sham group, while *P. copri* or GUO treatment increased their expression compared to that in the TBI group (Fig. 8K). Overall, *P. copri* transplantation help to attenuated BBB disruption in mice after TBI.

### *P. Copri* treatment attenuated neuronal apoptosis after TBI

**Fig. 9:**
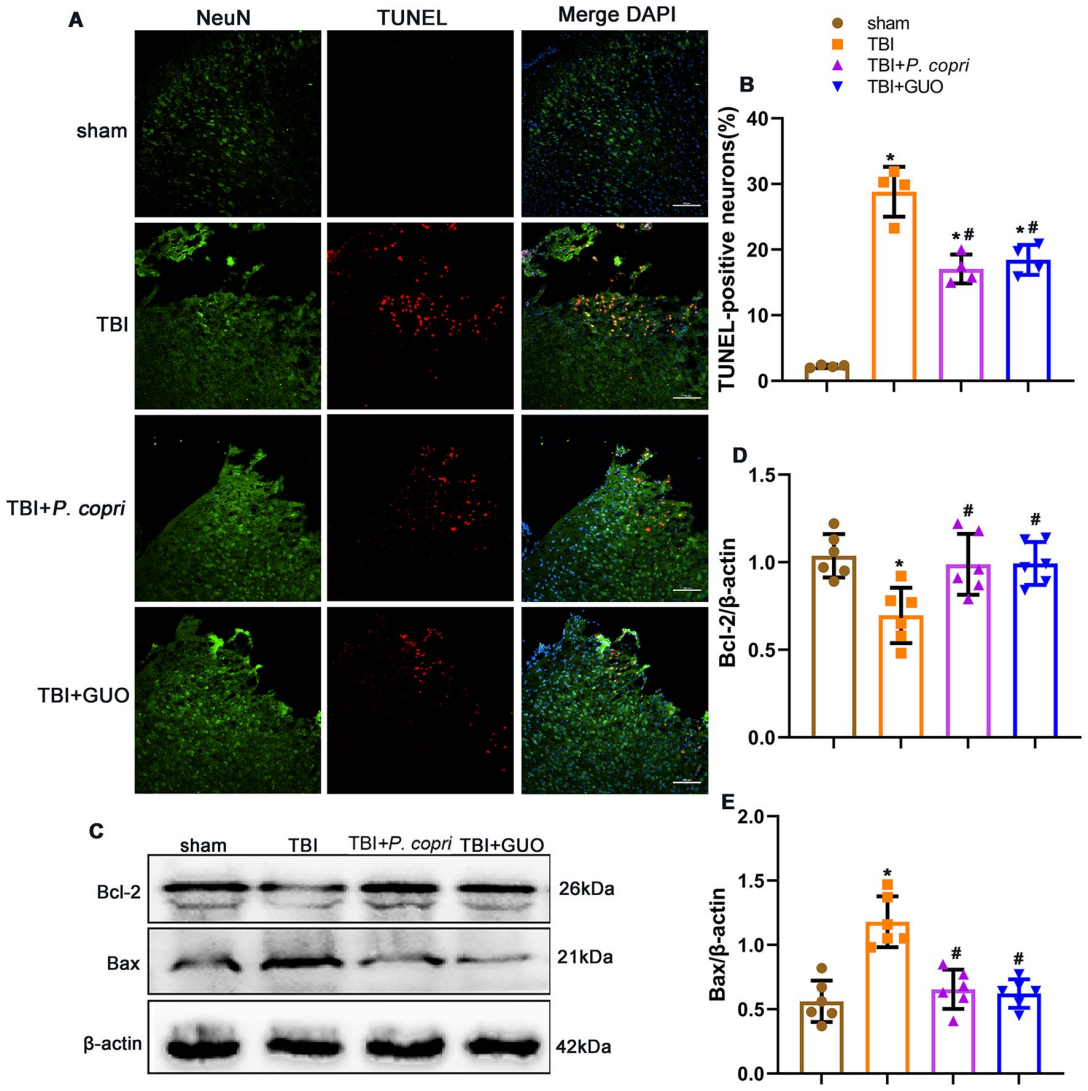
*P. copri* treatment attenuated neuronal apoptotic death and expression of apoptosis-related proteins after TBI. **A**. Representative images of dead cells (TUNEL, red) and neurons (NeuN, green) surrounding the lesion sites. Scale bar = 100 μm. **B**. Quantitative analysis of TUNEL-positive neurons surrounding lesion sites after TBI. *, *p* < 0.05 vs. sham; #, *p* < 0.05 vs. TBI, *n* = 4 per group. **C**. Representative Western blot bands of Bcl-2, Bax and β-actin at the lesion sites after TBI. **D**-**E**. Quantitative analysis of Bcl-2 (**D**) and Bax (**E**) density at the lesion sites after TBI. *, *p* < 0.05 vs. sham; #, *p* < 0.05 vs. TBI, *n* = 6 per group. One-way ANOVA was used to compare the means of different groups followed by a Tukey post hoc multiple-comparisons test

The TUNEL-positive neurons surrounding the trauma site was significantly increased after TBI. However, they were reduced in the TBI + *P. copri* group and TBI + GUO groups compared to the TBI group as shown by double immunofluorescence staining of TUNEL and NeuN (Fig. 9A and B). Furthermore, Western blotting was used to measure the expression of the apoptotic markers Bcl-2 and Bax after TBI. The results showed that the expression of Bcl-2 was significantly increased and the expression of Bax was significantly decreased with *P. copri* or GUO treatment when compared with the TBI group (Fig. 9C-E).

### The GUO-PI3K/Akt pathway was involved in the neuroprotective effects of *P. Copri* treatment

**Fig. 10:**
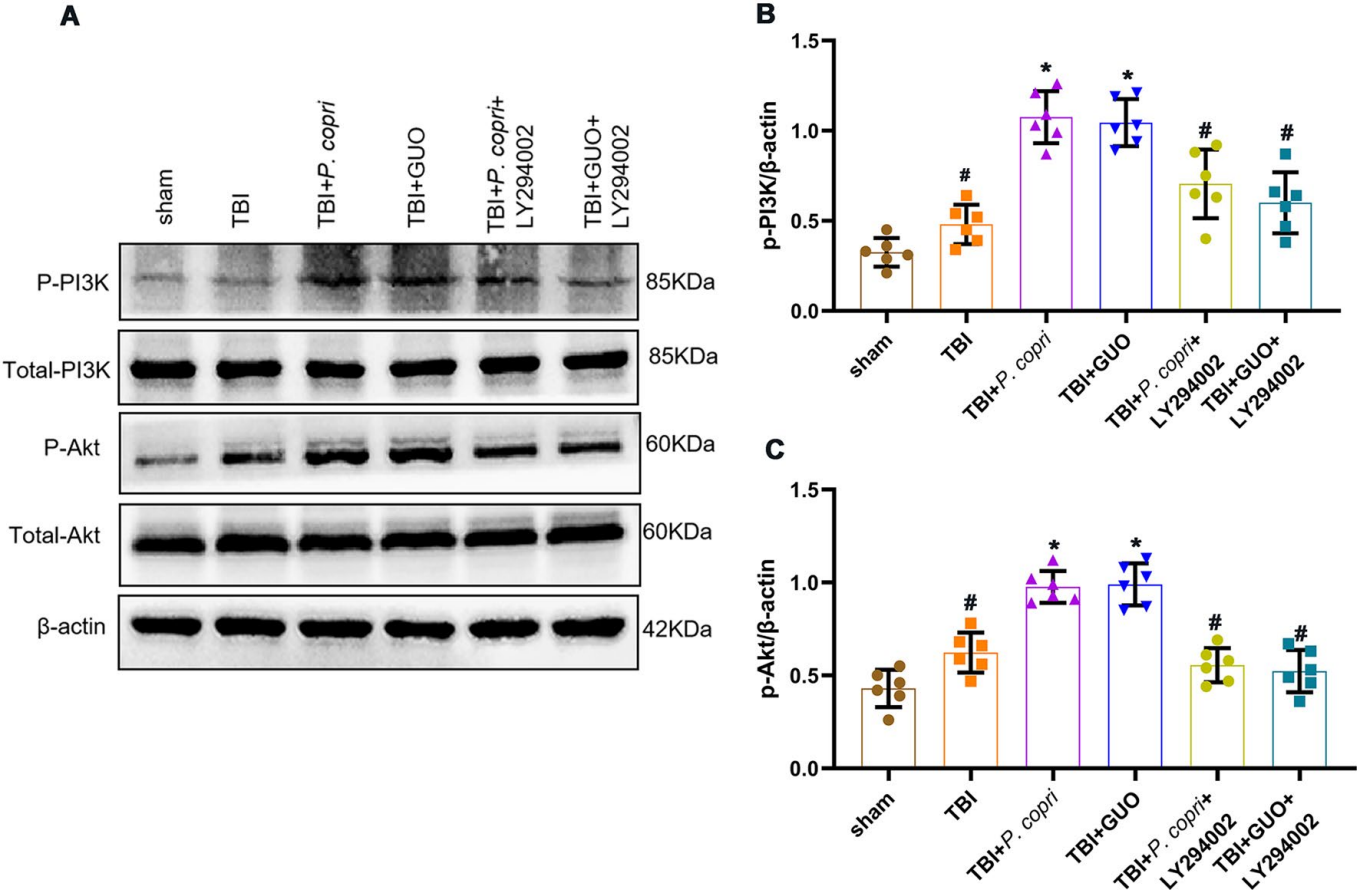
The GUO-PI3K/Akt axis was involved in the neuroprotective effects of *P. copri*. **A**. Representative Western blot bands of total PI3K, p-PI3K, total Akt, p-Akt and β-Actin at the lesion sites after TBI. **B**-**C**. Quantitative analysis of p-PI3K (**B**) and p-Akt (**C**) density at the lesion sites after TBI. *, *p* < 0.05 vs. sham; #, *p* < 0.05 vs. TBI + *P. copri* or TBI + GUO, *n* = 6 per group. One-way ANOVA was used to compare the means of different groups followed by a Tukey post hoc multiple-comparisons test

Previous research has verified that GUO can reduce oxidative stress after TBI by activating the PI3K/Akt pathway [25]. Therefore, we use Western blot analysis to examine the expression of PI3K/Akt pathway. The results showed that after *P. copri* or GUO treatment, the expression of p-PI3K and p-Akt significantly increased when compared with both the sham group and TBI group, and LY294002 inhibited the increase in p-PI3K and p-Akt expression caused by *P. copri* or GUO (Fig. 10A-C).

**Fig. 11:**
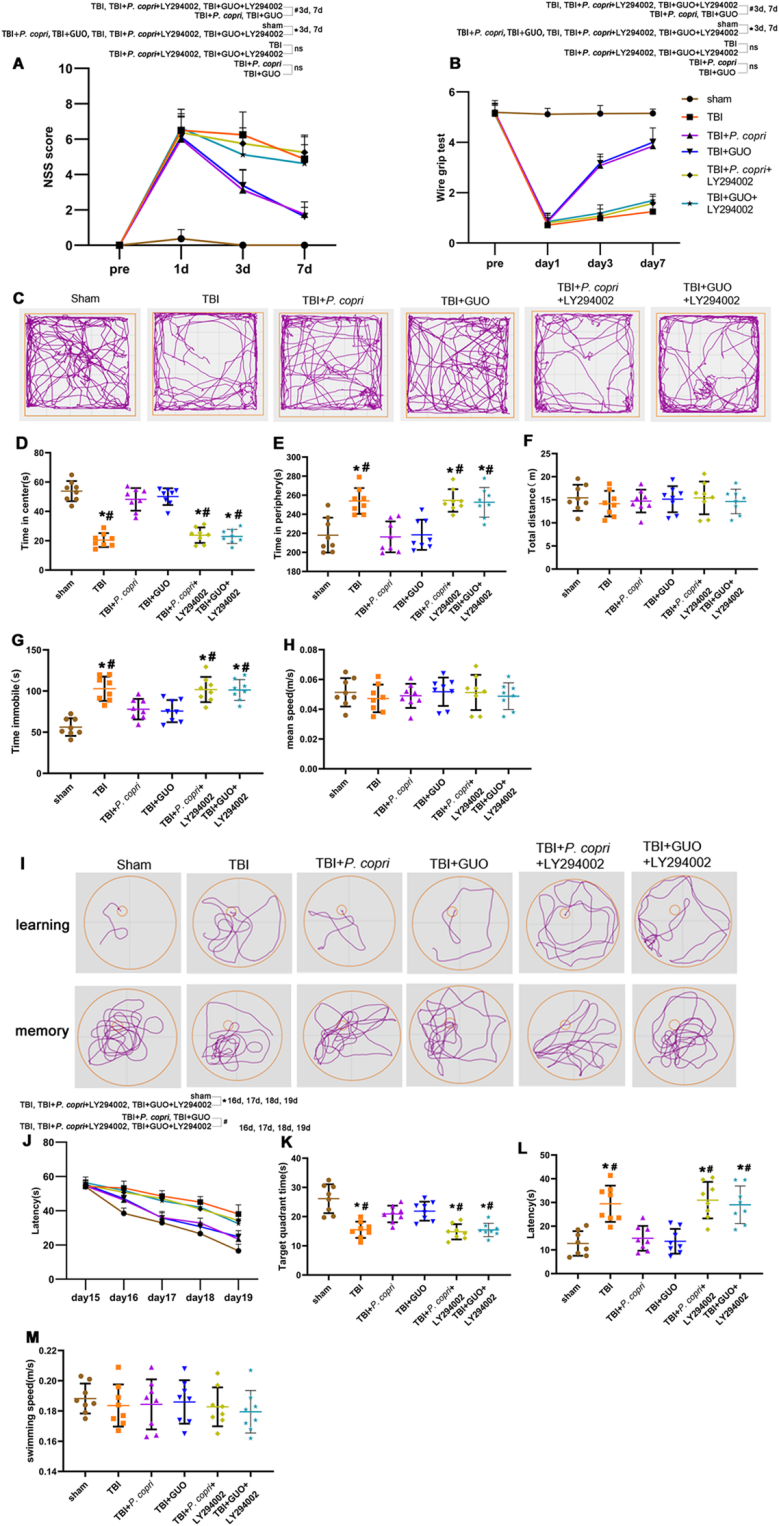
LY294002 inhibited the neuroprotective effects of *P. copri*. **A**-**B**. Quantitative analysis of short-term neurological function using NSS scores (**A**) and wire grip scores (**B**). *, *p* < 0.05 vs. sham; #, *p* < 0.05 vs. TBI + *P. copri* or TBI + GUO. *n* = 8 per group. **C**. Representative images of the movement path of mice in the open field test 14 days after TBI. **D**-**H**. Quantitative analysis of central time (**D**), periphery time (**E**), total distance (**F**), time immobile (**G**) and mean speed (**H**) travelled by mice in the open field test. *, *p* < 0.05 vs. sham; #, *p* < 0.05 vs. TBI + *P. copri* or TBI + GUO, *n* = 8 per group. **I**. Representative images of the swim path of mice in the Morris water maze. **J**-**M**. Quantitative analysis of latency in the learning test (**J**), the time in the target quadrant (**K**), the latency (**L**) and the average swimming speed (**M**) in the probe trial. *, *p* < 0.05 vs. sham; #, *p* < 0.05 vs. TBI + *P. copri* or TBI + GUO, *n* = 8 per group. Two-way repeated-measures ANOVA with Tukey’s post hoc multiple-comparisons test was used to analyze continuously measured data. One-way ANOVA was used to compare means of different groups followed by a Tukey post hoc multiple-comparisons test

Furthermore, the administration of LY294002 resulted in the inhibition of the decrease in NSS scores and enhancement in wire grip scores caused by *P. copri* or GUO (Fig. 11A-B). The OFT test showed the mice in the TBI + *P. copri* + LY294002 group and TBI + GUO + LY294002 groups exhibited more anxiety related behavior as opposed to those in the TBI + *P. copri* group and TBI + GUO group (Fig. 11C-H). Learning and memory function in mice was weakened in the TBI + *P. copri* + LY294002 group and TBI + GUO + LY294002 groups in comparison to the TBI + *P. copri* group and TBI + GUO groups, as evidenced by the results of Morris water maze test (Fig. 11I-M).

### The GUO-PI3K/Akt axis regulated the effects of *P. Copri* on oxidative stress, the blood‒brain barrier and neuronal apoptosis in TBI mice

**Fig. 12:**
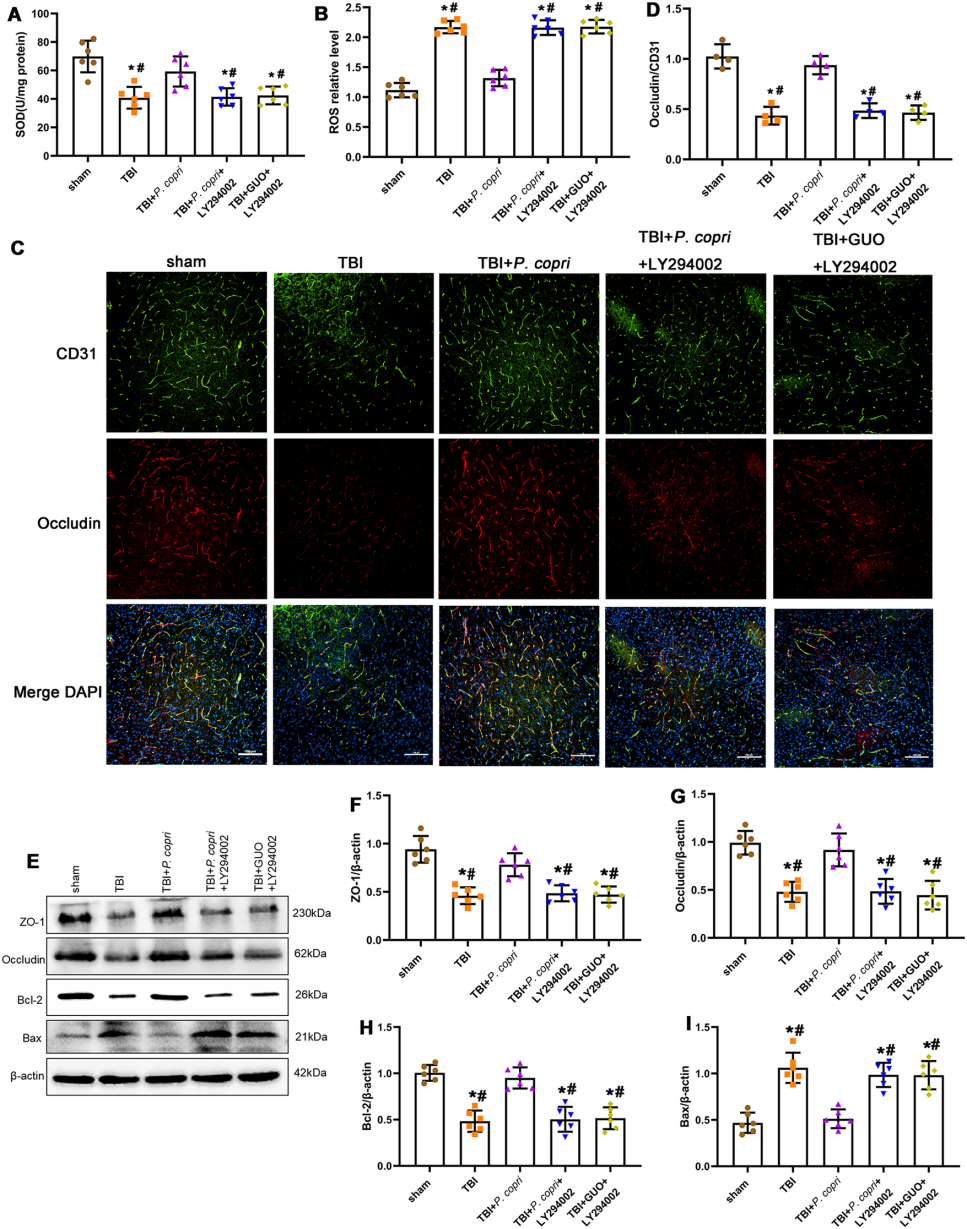
The GUO-PI3K/Akt axis was involved in the effects of *P. copri* on oxidative stress, blood-brain barrier and neuronal apoptosis in TBI mice. **A**-**B**. Oxidative stress after TBI. SOD activity (**A**) and ROS level (**B**). *, *p* < 0.05 vs. Sham; #, *p* < 0.05 vs. TBI + *P. copri*, *n* = 6 per group. **C**. Representative images of double immunofluorescence staining for Occludin and CD31, nuclei were stained with DAPI (blue). Scale bar = 100 μm. D. Quantitative analysis of relative Occludin fluorescence intensity in different groups. *, *p* < 0.05 vs. sham; #, *p* < 0.05 vs. TBI + *P. copri*, *n* = 4 per group. **E**. Representative Western blot bands of ZO-1, Occludin, Bcl-2, Bax and β-Actin at the lesion sites after TBI. F-I. Quantitative analysis of the relative density of ZO-1 (**F**), Occludin (**G**), Bcl-2 (**H**) and Bax (**I**). *, *p* < 0.05 vs. sham; #, *p* < 0.05 vs. TBI + *P. copri*, *n* = 6 per group. One-way ANOVA was used to compare means of different groups followed by a Tukey post hoc multiple-comparisons test

Compared with the sham group, all the experiment groups (including the TBI group, TBI + *P. copri* + LY294002 group and TBI + GUO + LY294002 group) displayed a significant decrease in SOD activity, with no statistical significance among them. Notably, the TBI + *P. copri* group showed a significant increase in SOD activity compared to the TBI group (Fig. 12A). At the same time, compared to the sham group, higher levels of ROS were measured in the TBI group, TBI + *P. copri* + LY294002 group and TBI + GUO + LY294002 group, while the TBI + *P. copri* group showed significantly reduced ROS compared to the TBI group (Fig. 12B).

In addition, LY294002 prevented the improvement of *P. copri* on loss of Occludin after TBI (Fig. 12C and D). Western blot assays revealed that LY294002 inhibited the increase in ZO-1 and Occludin expression (Fig. 12E-G). We examined Bcl-2 and Bax expression by western blot to determine the effect of LY294002 on the apoptosis. The results showed that LY294002 inhibited the increase in Bcl-2 expression and the decrease in Bax expression caused by *P. copri* (Fig. 12E, H and I).

## Discussion

Numerous studies have confirmed changes in the gut microbiota after TBI that can produce a series of effects on the central nervous system through the brain-gut axis [13, 48–50]. It has been reported that *Prevotella* changes significantly after TBI, in which *P. copri* bacteria are significantly reduced [15, 51], but it has not been reported whether.

*P. copri* has an effect on the pathophysiology of TBI. In our study, we found that *P. copri* experienced a continuous decline for 7 days after TBI, which was consistent with reports in the literature. We found that *P. copri* treatment improved neurological dysfunction after TBI, including learning memory function and anxiety related behaviors in TBI mice. *P. copri* transplantation helps recover dysbiosis of the gut microbiota after TBI and increases the level of guanosine. Furthermore, we proved that *P. copri* transplantation relieved neurological deficits possibly through the GUO-PI3K/Akt signaling pathway.

The safety of *P. copri* transplantation in normal mice has been proved by Phebe Verbrugghe et al [30]. They found that histological analysis of liver, pancreas, colon, heart, lung, spleen, kidney, testis, brain and epididymal fat showed no microscopic abnormalities after a long period (for 29 consecutive days) *P. copri* oral administration. Repeated-dose oral administration of *P. copri* did not result in any significant changes in the blood cell count or serum biochemistry parameters. On the basis of this valuable study, we discovered more roles for *P. copri*. We found that *P. copri* could improve intestinal permeability and intestinal motility and reshape the gut microbiota of TBI mice. Gastrointestinal motility contributes to the maintenance of a healthy intestinal barrier as it clears luminal debris and, importantly prevents the proliferation of microbiota. Impaired motility can potentially contribute to bacterial dysbiosis in TBI, which may have deleterious effects on gut barrier integrity [45]. Besides, the FITC-dextran assay was used in this study as a measure of intestinal permeability. The gut-transit time affects the speed of movement of the FITC-dextran along the digestive tract [52]. FITC-dextran reached its maximal concentration in the plasma of adult wildtype C57BL/6 mice 4 h after its oral administration. With a physiologic gut-transit time, FITC-dextran can reach the colon within 4 h [53]. However, a recent study in adult wildtype C57BL/6 mice reported that FITC-dextran reached its maximal concentration in plasma within the first 2 h after oral administration [54]. Therefore, measuring the plasma concentration of FITC-dextran at only one time point cannot accurately assess the site of intestinal barrier dysfunction. Thus, we used the concentration of FITC-dextran in plasma only as a measure for the intestinal permeability assay. Together with the results of tight junction proteins in the intestinal wall, it is well convinced that *P. copri* treatment can improve intestinal permeability and motility in TBI mice.

Through 16 S rDNA sequencing, we found that *P. copri* treatment increased *Akkemansia*, *Lactobacillus*, *Lachnospiraceae* and *Lachnospiraceae-NK4A136.* As an important genus of intestinal bacteria, *Akkermansia* degrades mucin and enhances gut barrier function [55]. *Lactobacillus*, *Lachnospiraceae* and *Lachnospiraceae-NK4A136* bacteria in the gastrointestinal tract produce butyrate and other short-chain fatty acids (SCFAs) by hydrolyzing starch and other sugars, which directly interact with the host’s immune system and regulate the surrounding microbial environment [56]. SCFAs are not only the main energy source of the colon but also responsible for intestinal epithelial protection and the regulation of inflammatory intestinal responses, favoring mucus synthesis and upregulating tight junction proteins [57]. Previous study mentioned that Prevotella can ferment dietary fiber to produce the SCFA acetate [17] and metabolize dietary fiber and fat to produce succinate [18]. However, we did not find a direct relationship between *P. copri* and SCFAs alterations in the serum metabolomics (Fig. 5). Therefore, the relationships between SCFAs and *P. copri* transplantation in TBI may require further detection over time.

In a study encompassing both humans and mice, *P. copri* was shown to enhance the ability to utilize complex polysaccharides, potentially by increasing glycogen storage [58]. In our study, we interestingly found that guanosine (GUO) expression was significantly increased after *P. copri* treatment. It has been reported that the gut microbiota is one of the contributors to guanosine nucleotide biosynthesis, such as *Akkermansia* [59] and *Lachnospiraceae* [60]. The increase in GUO might be related to the change in intestinal flora. That is to say, *P. copri* treatment may not only cause the remodeling of bacterial flora but also directly lead to an increase in GUO. A large number of studies have confirmed that GUO could reduce oxidative stress, inflammation and neuronal apoptosis after TBI and improve the destruction of the blood brain barrier after TBI [61]. GUO is an endogenous neuroprotective nucleoside that provides the long-term benefits in controlling brain neurodegeneration, mainly due to its capacity to activate the antioxidant defense system and maintain the redox system [61]. Intraperitoneal administration of GUO increases GUO of cerebrospinal fluid levels approximately two to three times in 30 min [62]. GUO is effective in reducing ROS generation [63] and preventing the collapse of mitochondrial membrane potential in hippocampal slices subjected to oxygen glucose deprivation [64]. In our study, treatment with *P. copri* or GUO ameliorated oxidative stress, repaired blood-brain barrier disruption, and reduced neuronal apoptosis in TBI mice. Therefore, the neuroprotective effect of *P. copri* treatment on TBI mice is firmly related to the increase in GUO.

Previous studies have confirmed that GUO can reduce oxidative stress after TBI by activating the PI3K/Akt pathway. GUO can reduce the excessive production of ROS by activating the PI3K/Akt pathway in astrocytes and hippocampal tissue in vitro [25]. GUO also alleviates mitochondrial oxidative stress injury caused by inhibition of the mitochondrial complex by activating the PI3K/Akt pathway in neuroblast cells [26]. In our experiment, we detected the key proteins in the PI3K/Akt pathway and found that the two proteins were significantly increased after *P. copri* treatment. Meanwhile, a PI3K inhibitor (LY294002) significantly inhibited the neuroprotective effect of *P. copri* and suppressed the effects of *P. copri* on oxidative stress, the blood-brain barrier and neuronal apoptosis in TBI mice. It can be suggested that *P. copri* transplantation causes GUO elevation, which activates the PI3K/Akt pathway and ultimately ameliorates neurological dysfunction in TBI mice.

TBI is a severe injury, and there is no effective therapeutic method that can be used in the current clinical scenario. In recent years, certain progress has been made in research on the brain-gut axis [65, 66]. The neuroprotective effect of fecal transplantation on TBI has also been confirmed by the literature [14, 67]. In our study, we used the common intestinal bacteria in the genus *Prevotella* (*P. copri*) for gavage treatment to further investigate the role of a specific bacterial species in TBI. There is still great controversy about the role of *P. copri* in human health. Even in the same disease, two opposite results have appeared [31]. There may be many reasons for this, such as the different diets of subjects in different experiments and the large differences between *P. copri* strains. All these factors affect the pathophysiological role of *P. copri* in the development of diseases. We believe that it is through the synergistic effect of multiple targets that *P. copri* can exert a beneficial action on TBI mice.

In this study, we have demonstrated for the first time that *P. copri* can promote the recovery of neurological function after TBI, however, there are several limitations in this study. First, antibiotics pretreatment was used to remove intestinal flora and minimize the gut microbiome difference between the experimental groups in a large number of studies. However, recently published study find that microbiome depletion prior to brain injury may exacerbate secondary inflammatory cascades [68]. Therefore, to further elucidate the impact of microbial alterations on the repair of brain injury, we suggest that an integrated approach to simultaneously avoid the secondary injury should be explored in future studies. Meanwhile, TBI patients will not likely have a depleted microbiome or antibiotics pretreatment upon injury in reality. In the future study, *P. copri* transplantation study without prior microbiome manipulation should be considered. Another limitation is that the research is carried out in male mice. However, sex plays a significant role in the outcome of TBI [69]. Female mice have higher ROS activities in the brain in the acute phase [70]. Glial cells in female mice are also less activated in the subacute phase of brain injury [71].Meanwhile, recent studies also found that post-CCI neurological complications may be influenced by the differential gut microbiota perturbation in a gender-dependent manner [72]. Thus, in order to minimize the confounding factors caused by gender, only single-sex mice were used in this study. In the future preclinical studies, mice of both sexes must be included for further in-depth studies. On the other hand, we used CCI model to mimic TBI in vivo, and craniotomy is an essential step in the CCI procedure. There is evidence that the craniotomy is not atraumatic and the effects of surgery should be controlled [73]. However, recently in vivo study of CCI model showed that no pathology was present in sham operated mice (craniotomy only) by MRI scan [74]. To mitigate these concerns to some extent, we included the craniotomy surgical sham groups as control in our study.

## Conclusions

In conclusion, the current study showed that *P. copri* transplantation can improve GI functions and remodel the gut microbiota after TBI. *P. copri* transplantation relieved the blood-brain barrier damage and reduced neuronal apoptosis through elevating GUO and activating the PI3K/Akt pathway. As a result, *P. copri* transplantation could relieve the neurological deficits, exerts an antioxidant effect.

### Electronic supplementary material

Below is the link to the electronic supplementary material.

Supplementary Material 1

## Data Availability

All data generated or analyzed during this study are included in thispublished article and its supplementary files.

## Abbreviations

ANOVA: Analysis of variance
APES: 3-Aminopropyl-triethoxysilane
BBB: Blood-Brain Barrier
Bax: BCL2-Associated X protein
Bcl-2: B cell lymphoma/leukemia-2 gene
CNS: Central nervous system
CAT: Catalase
CCI: Controlled Cortical Impact
DAPI: 4’,6-diamidino-2-phenylindole
DMB: 3,3-dimethyl-1-butanol
EB: Evans blue
ECs: Endothelial cells
ECL: Electro-chemi-luminescence
GSSG: Oxidized glutathione
GSH: Reduced glutathione
GM: Gut Microbiome
IF: Immunofluorescence
MGBA: Microbiome-Gut-Brain Axis
MWM: Morris water maze test
NSS: Neurological Severity Score
OFT: Open field test
PVDF: Polyvinylidene Fluoride
PFA: Paraformaldehyde
*P. copri*: *Prevotella copri*
PBS: Phosphate buffer saline
PAGE: Polyacrylamide gel eletrophoresis
ROS: Reactive oxygen species
SDS: Sodium dodecyl sulfate
SOD: Superoxide dismutase
TBI: Traumatic brain injury
TJ: Tight junction
TME: Transmission electron microscope
WB: Western blot
ZO-1: Zona Occludens 1
