# Supplementary material for: Prevotella copri transplantation promotes neurorehabilitation in a mouse model of traumatic brain injury
Source: J Neuroinflammation. 2024 Jun 4;21:147. doi: 10.1186/s12974-024-03116-5 (PMC11151605; doi:10.1186/s12974-024-03116-5)
Supplement: Supplementary file 1 — Supplementary Material 1 [file 12974_2024_3116_MOESM1_ESM.docx]

**Supplemental Materials**

***Prevotella copri* transplantation promotes neurorehabilitation in a mouse model of traumatic brain injury**

**Supplemental methods**

**Supplemental Figures and Figure Legends**

**Supplemental methods**

**Table1 Neurological severity score (NSS) for head-injured mice**

| **Task** | **NSS** |
| --- | --- |
| Presence of mono- or hemiparesis | 1 |
| Inability to walk on a 3-cm-wide beam 1 | 1 |
| Inability to walk on a 2-cm-wide beam | 1 |
| Inability to walk on a 1-cm-wide beam | 1 |
| Inability to balance on a 1-cm-wide beam | 1 |
| Inability to balance on a round stick (0.5 cm wide) | 1 |
| Failure to exit a 30-cm-diameter circle (for 2 min) | 1 |
| Inability to walk straight | 1 |
| Loss of startle behavior | 1 |
| Loss of seeking behavior | 1 |
| Maximum total | 10 |

**One point is awarded for failure to perform a task.**

**Suppl File 1** Detailed procedures of Real time quantitative PCR (qPCR)

The qPCR cycling conditions were as follows: pre-denaturation of 95 °C for 3 min, 40 cycles of denaturation of 95 °C for 5 s, annealing step at 58 °C for 30s and extension time at 72℃ for 1min. PCR reactions were performed in triplicate 20μL mixture containing 10μL 2X ChamQ SYBR Color qPCR Master Mix, 0.8μL of each primer (5 μM), 0.4μL 50 X ROX Reference Dye 1, 2μL template DNA, 6μL deionized distilled water for a total volume of 20 μL. qPCR was performed with ABI7300 fluorescent qPCR instrument (Applied Biosystems, USA). Genomic DNA from *P. copri* was used to generate a standard curve to quantitate ng of *P. copri* present per mg of total feces.

**Supplemental Figures and Figure Legends**

**
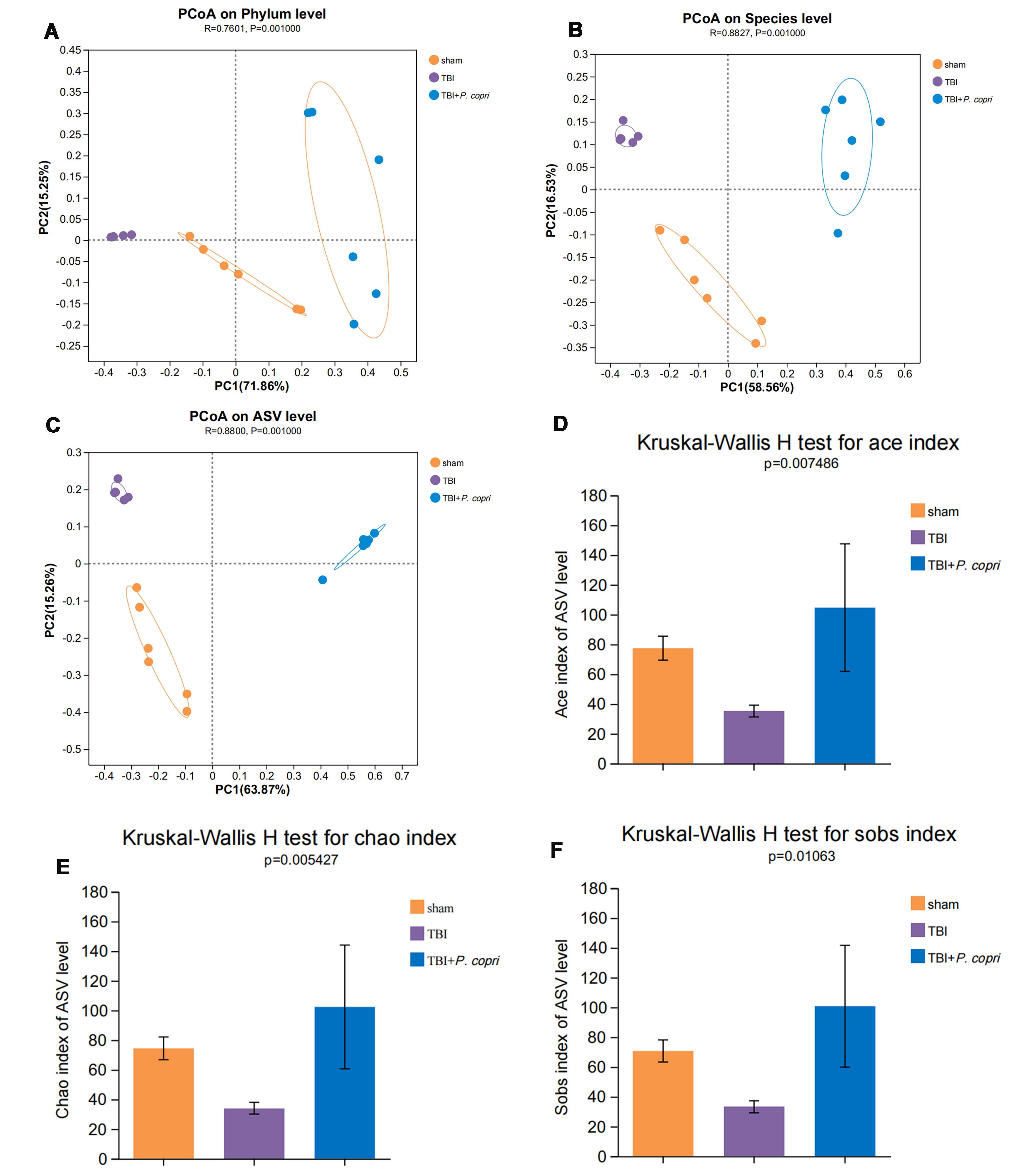
**

**Suppl Fig. 1 *P. copri* treatment reshaped the gut microbiota of TBI mice.** A-C. PCoA of gut microbiome composition at the level of phylum (A), species (B) and ASV (C) based on Bray-Curtis distance. D-F. Indices of α-diversity measured by Ace(D), Chao(E) and Sobs(F).


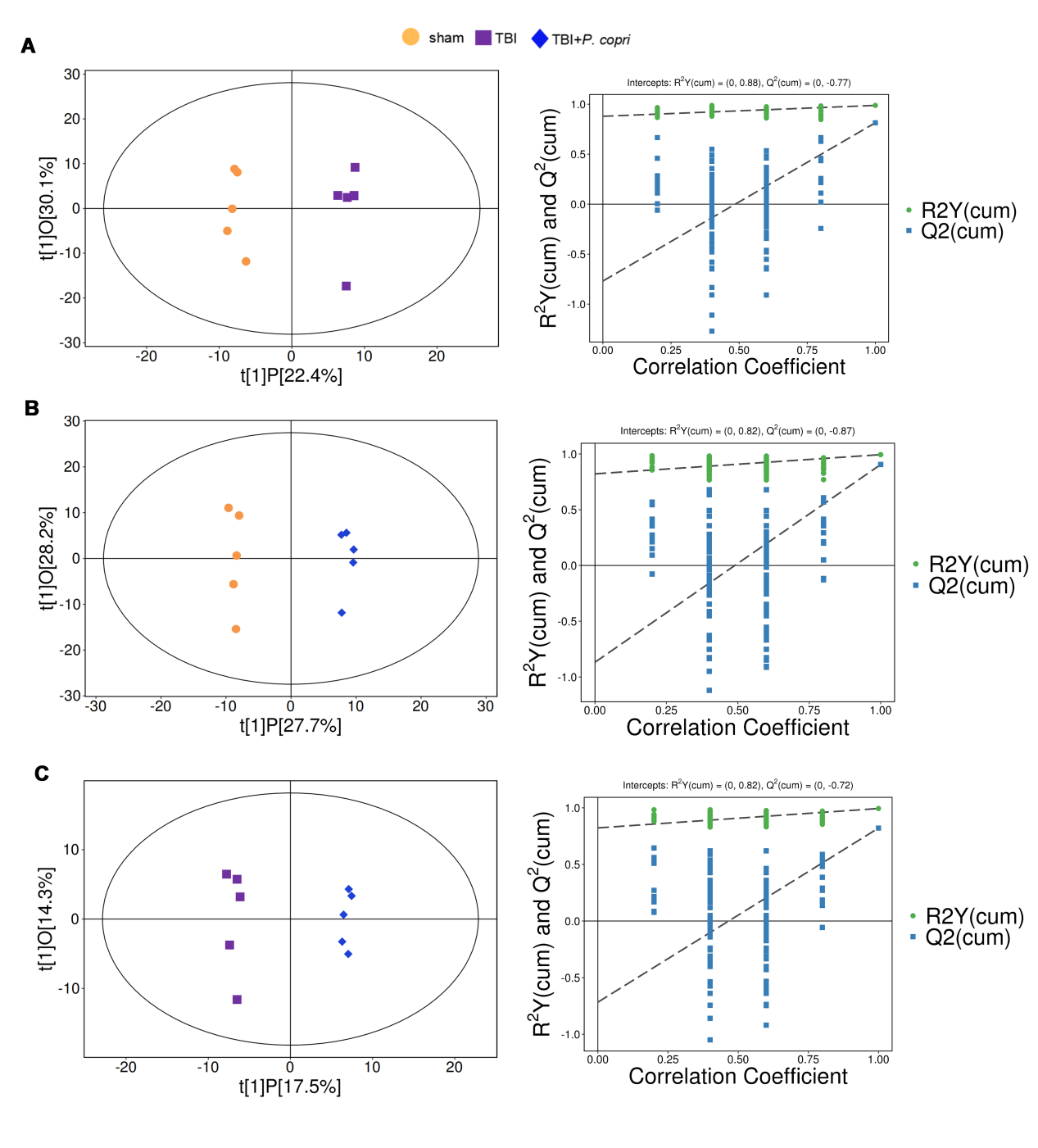


**Suppl Fig. 2 OPLS-DA model and permutation test of the three groups in serum metabolomics.** A. sham vs TBI. B. sham vs TBI+*P. copri*. C. TBI vs TBI+*P. copri*

**
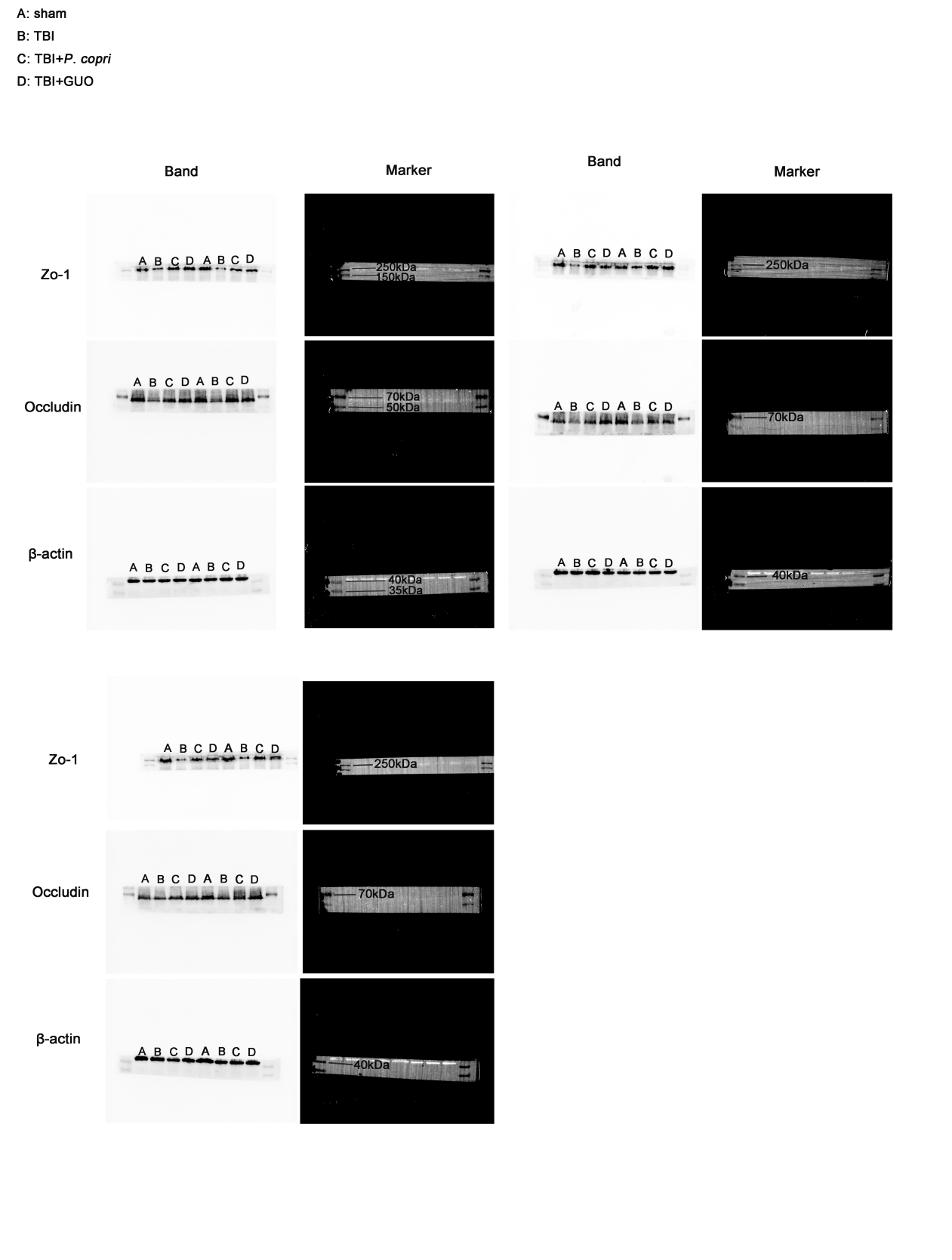
**

**Suppl Fig. 3** Raw western blot bands in Fig. 8G-I

**
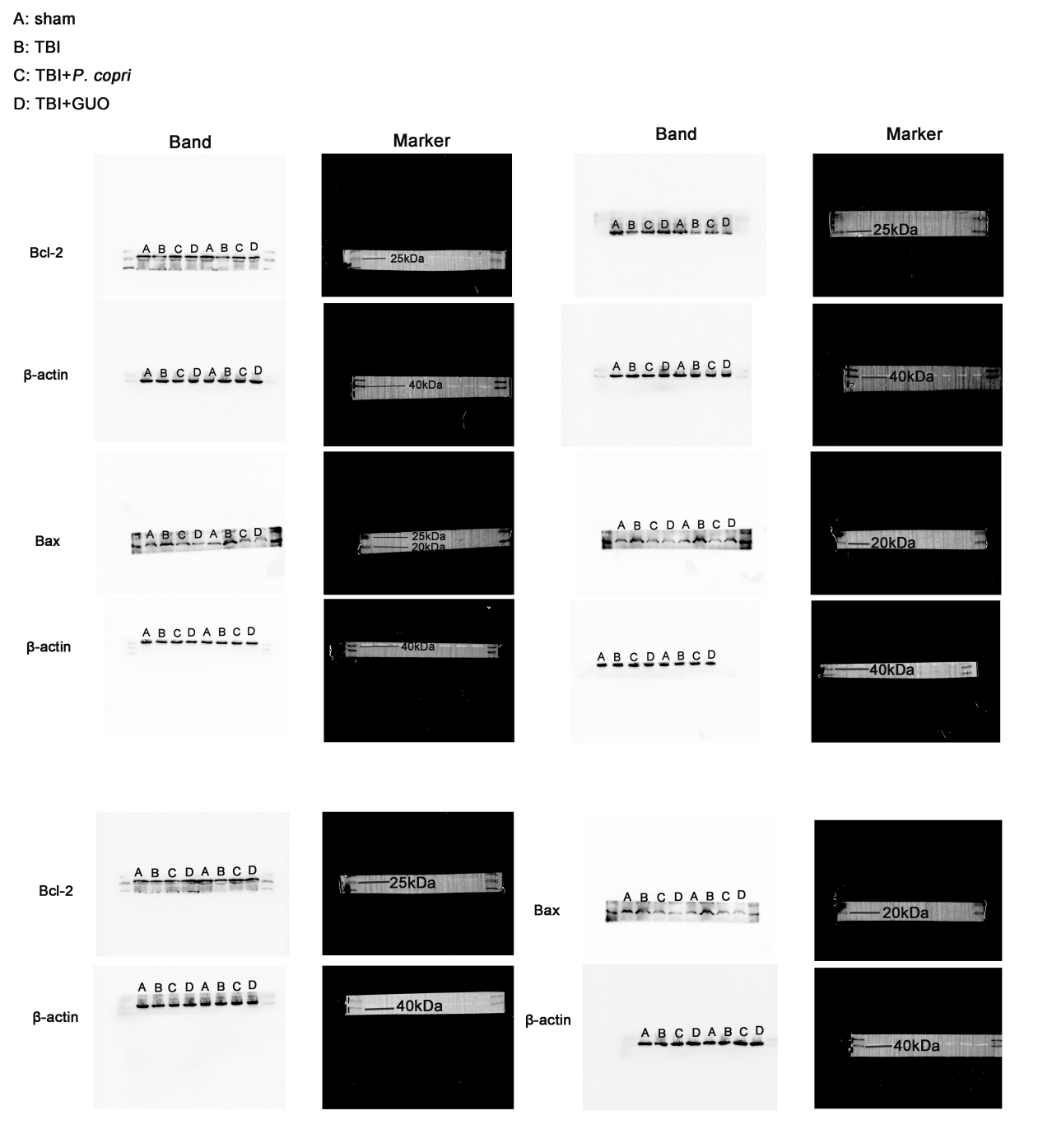
**

**Suppl Fig. 4** Raw western blot bands in Fig. 9C-E

**
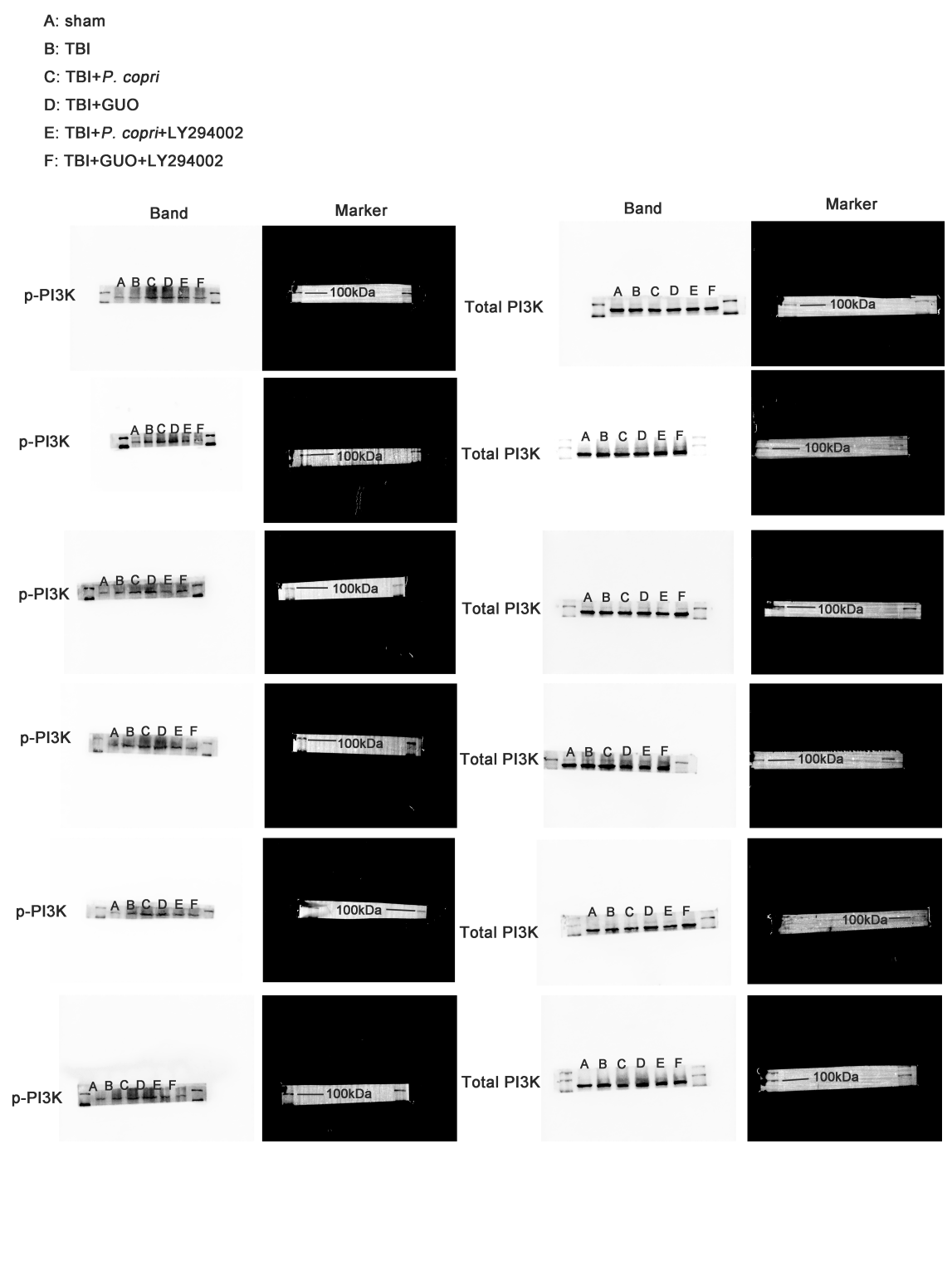
**

**Suppl Fig. 5** Raw western blot bands in Fig. 10A and B

**
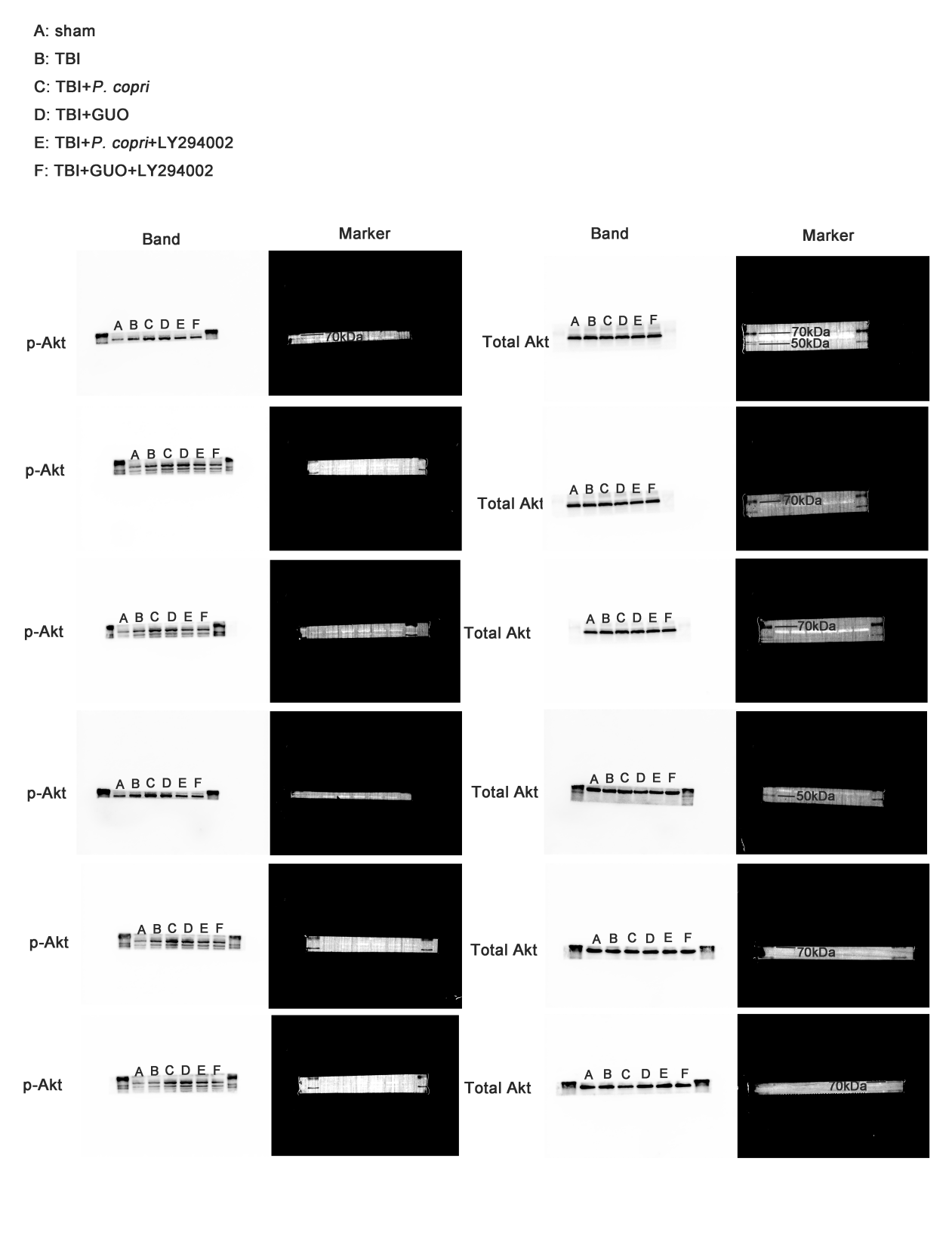
**

**Suppl Fig. 6** Raw western blot bands in Fig. 10A and C

**
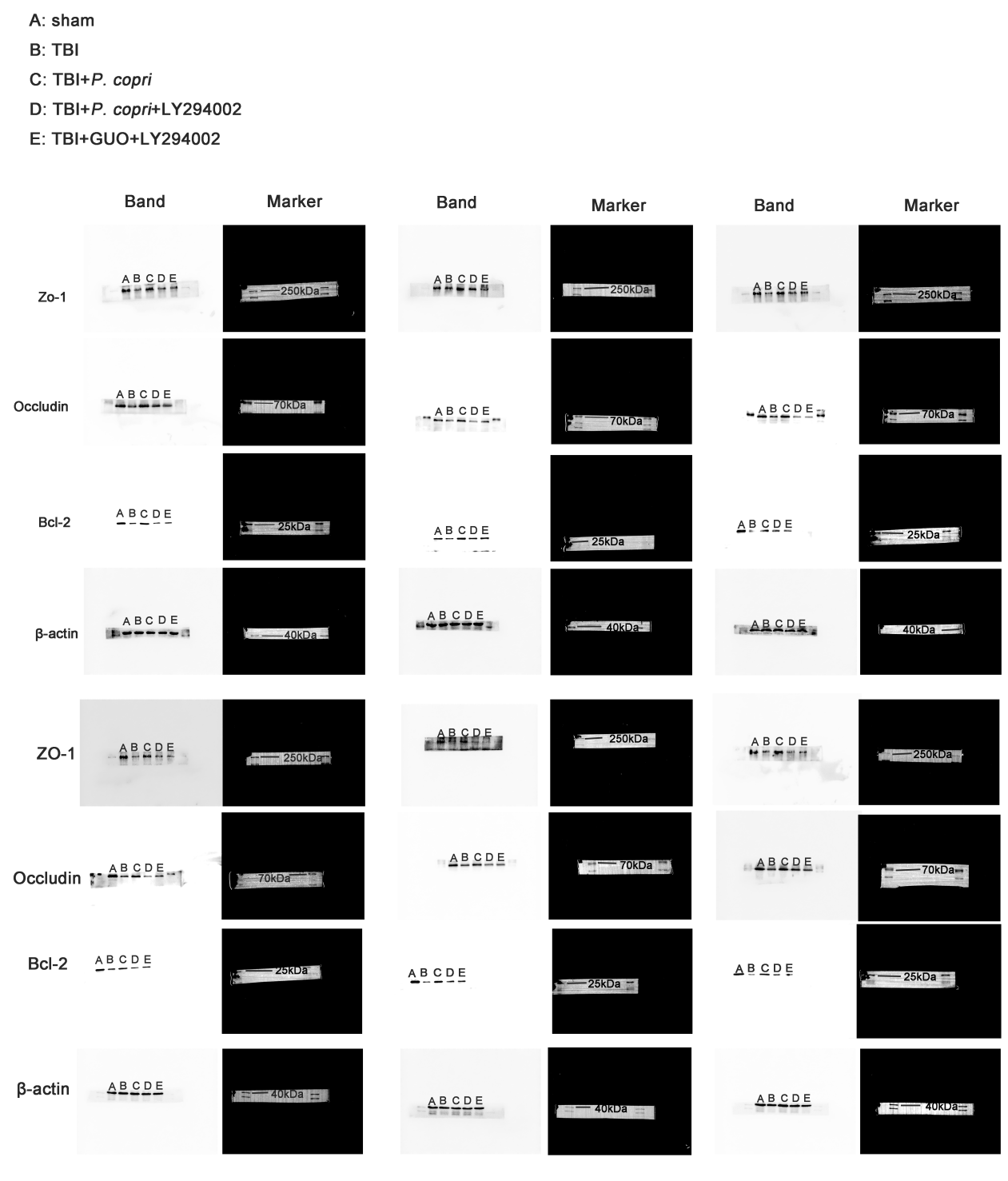
**

**Suppl Fig. 7** Raw western blot bands in Fig. 12E-H

**
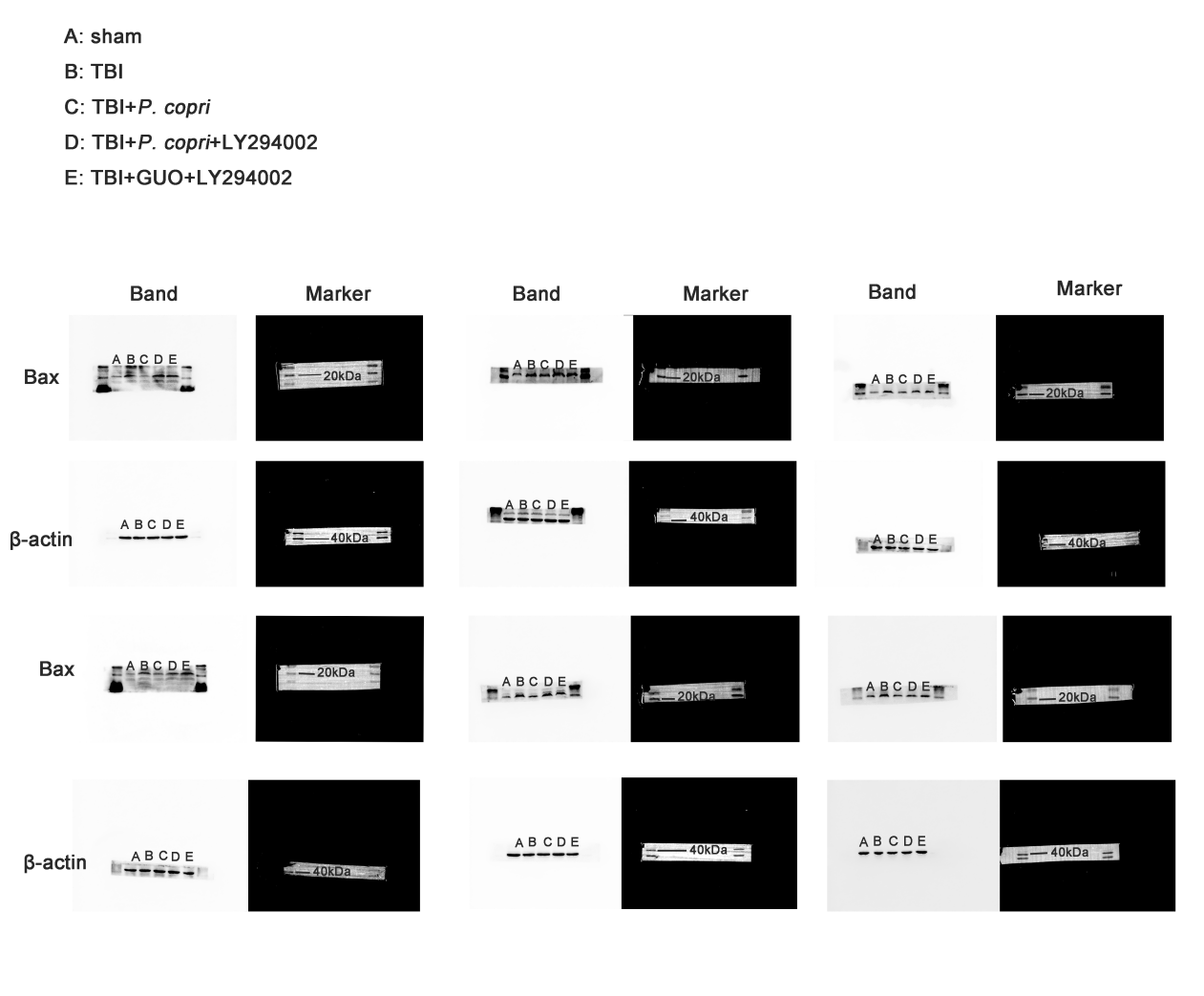
**

**Suppl Fig. 8** Raw western blot bands in Fig. 12E and I
